# Identification of non-ribosomal peptide synthetase in *Ganoderma boninense* Pat. that was expressed during the interaction with oil palm

**DOI:** 10.1038/s41598-021-95549-8

**Published:** 2021-08-11

**Authors:** Neda Shokrollahi, Chai-Ling Ho, Nur Ain Izzati Mohd Zainudin, Mohd As’wad Bin Abul Wahab, Mui-Yun Wong

**Affiliations:** 1 Department of Plant Protection, Faculty of Agriculture, Universiti Putra Malaysia, 43400 Serdang, Selangor Malaysia; 2 Department of Cell and Molecular Biology, Faculty of Biotechnology and Biomolecular Sciences, Universiti Putra Malaysia, 43400 Serdang, Selangor Malaysia; 3 Department of Biology, Faculty of Science, Universiti Putra Malaysia, 43400 Serdang, Selangor Malaysia; 4 Institute of Plantation Studies, Universiti Putra Malaysia, 43400 Serdang, Selangor Malaysia

**Keywords:** Fungi, Effectors in plant pathology, Plant immunity, Agricultural genetics

## Abstract

Basal stem rot (BSR) of oil palm is a disastrous disease caused by a white-rot fungus *Ganoderma boninense* Pat. Non-ribosomal peptides (NRPs) synthesized by non-ribosomal peptide synthetases (NRPSs) are a group of secondary metabolites that act as fungal virulent factors during pathogenesis in the host. In this study, we aimed to isolate NRPS gene of *G. boninense* strain UPMGB001 and investigate the role of this gene during *G. boninense*-oil palm interaction. The isolated NRPS DNA fragment of 8322 bp was used to predict the putative peptide sequence of different domains and showed similarity with *G. sinense* (85%) at conserved motifs of three main NRPS domains. Phylogenetic analysis of NRPS peptide sequences demonstrated that NRPS of *G. boninense* belongs to the type VI siderophore family. The roots of 6-month-old oil palm seedlings were artificially inoculated for studying NRPS gene expression and disease severity in the greenhouse. The correlation between high disease severity (50%) and high expression (67-fold) of *G. boninense* NRPS gene at 4 months after inoculation and above indicated that this gene played a significant role in the advancement of BSR disease. Overall, these findings increase our knowledge on the gene structure of NRPS in *G. boninense* and its involvement in BSR pathogenesis as an effector gene.

## Introduction

Oil palm (*Elaeis guineensis* Jacq) is known as a native tree from West Africa^1,2^. The oil palm industry plays a prominent role in Malaysia's economic growth^3^. In 2019, Malaysia was known as the second-largest producer and exporter of palm oil in the world^4^. Basal stem rot (BSR) is a destructive disease in the oil palm plantations of South East Asia (SEA), particularly, Malaysia and Indonesia that are responsible for up to 90% of the world's palm oil export^5^. The disease is caused by a white-rot fungus, *Ganoderma boninense* Pat. which has threatened the oil palm industry for more than eight decades without any effective treatment available until now^5^ even though several methods have been implemented to control the disease including cultural practices (elimination of diseased residues, ploughing and fallowing before farming, planting of legume cover crops and effective fertilizers management), mechanical soil mounding and surgical removal of basidiocarps, utilization of fungicides and biological control using *Trichoderma* spp. and *Pseudomonas aeruginosa*^6^. Therefore, a better understanding of Ganoderma-oil palm interaction is essential to develop effective management strategies^7^ such as the identification of genes related to secondary metabolites secreted by *G. boninense* during its interaction with oil palm^8^.

Secondary metabolites (SMs) consist of low-molecular-weight compounds that unlike primary metabolites are not usually regarded as a crucial factor for life while they have various roles^8,9^. These natural products are synthesized by specific fungal taxon, mostly by filamentous fungi such as Pezizomycotina in Ascomycete class and many Basidiomycete classes including Agaricomycetes and Exobasidiomycetes, and unanticipated taxa like *Kluyveromyces lactis* (a Kluyveromyces yeast) that was recently found in the pulcherrimin gene cluster^10^. Among the secondary metabolites, non-ribosomal peptide synthetases (NRPSs) have been the focus of study in recent years due to their role in biological processes including biotic stress responses^11^. Non-ribosomal peptides (NRPs) are fungal secondary metabolites whose biosynthesis is independent of ribosomes and mRNA but synthesized by NRPSs^12^. NRPS-derived metabolites show a wide chemical variety such as incorporating proteinogenic and non-proteinogenic amino acids in their D- and L- configurations by multi-domain, multi-modular enzymes^13^. l-δ-(α-Aminoadipoyl)-l-cysteinyl-d-valine synthetase (ACVS), enniatin synthetase, cyclosporine synthetase, HC-toxin synthetase (HTS1), and bassianolide synthetase are some examples of NRPSs of plant pathogenic fungi^12,14^. The genome of many pathogenic fungi was found to contain NRPS genes which are now available on publicly available databases such as https://mycocosm.jgi.doe.gov/pages/sm-clusters-summary.jsf^15^.

Elicitors act as bioactive signals that are recognized by pattern recognition receptors (PRR) of plants. In the first level of inducible defense strategy against pathogens, PRRs, which initiate defense responses known as (pathogen-associated molecular patterns) PAMP-triggered immunity (PTI), caused accumulation of reactive oxygen species (ROS) in the plasma membrane^16^. For example, PebC1 (as an elicitor) caused a defense reaction in tomato against *Botrytis cinerea*^17^. The pathogen invasion is successful by secretion of various race-specific elicitors (effectors) directly into the plant cells, which inhibits PTI activation in the plant. In the second plant protection strategy, effector-triggered immunity (ETI) leads to expressed defense genes for the prevention of progression of the disease avirulence (*avr*) genes, from the aggressive pathogen, encode effectors corresponding to the resistance (*R*) genes in its specific host. If the fungal strain has a virulent form of avr effector, R protein is no longer able to detect the pathogen, so ETI process would be stopped^16^.

Virulent fungi destroy their host plants by various strategies including the secretion of SMs such as toxins. Some necrotrophic fungi have specific effector proteins which are active against their specific hosts, known as host-specific toxins (HSTs). HST secretion is pivotal for enhancing the pathogenicity and virulence in the host^16^. The target of toxin effectors in necrotrophic fungi is one of the main signaling or regulatory pathway that triggers gene-mediated resistance or the decrease in the regulation of defense enzymes; thereby, reducing the host sensitivity boosts against fungal attack^18^. Effectors are produced by virulent pathogens to enhance infection in the host plants^19^. To illustrate, HTS1 is an effector produced by *Cochliobolus carbonum* Race 1 during the pathogenicity in the susceptible genotype of *hmhm* maize^20^. This fungal effector disturbed the regulatory system of the plant by blocking calmodulin signalling or histone acetylation^18^. In contrast, *Hm1* and *Hm2* genes in maize are responsible for encoding carbonyl reductases that function to suppress the toxin; however, they are not successful^20^. A study on avirulence effector genes in *Magnaporthe oryzae* (the causal agent of rice blast) interacting with rice revealed that *ACV1* gene (hybrid polyketide synthase (PKS)–NRPS genes) acts against the *R* gene *(Pi33*) in rice. Although the exact determination of the function of these metabolites was complicated, *ACV1* expression was simultaneously linked to the appressorium-mediated penetration in the cuticle of rice^21,22^. Another example of a toxin effector produced by NRPS gene is AM-toxin that encoded by *AMT1* gene in the apple pathotype of *Alternaria alternata* during the interaction with the host^23^. AM-toxin has two attack sites including disordering in the chloroplast’s membrane by a reduction of chlorophyll substance and inhibition of photosynthetic CO_2_ and damaging effect in the plasma layer^24^.

A canonical module of NRPS enzyme is composed of three different standard domains: adenylation (A), thiolation (T) or peptidyl carrier protein (PCP), and condensation/peptide-bond formation (C) which is recognized as one NRPS module^25^. Adenylate forming domain (AFD_class_I) superfamily belongs to the group of acyl- and aryl-CoA ligases, adenylation domain of NRPS^12^. The adenylate-forming enzymes catalyze an ATP-dependent in two steps. In the first step, a carboxylate substrate will be activated as an adenylate and in the next step, the carboxylate will be transferred to the pantetheine group which is a coenzyme A or an acyl-carrier protein^12^. A set of conserved sequence motifs (A1–A10) has structural and functional roles for diagnosis and binding of the substrates, which is specific in A domain^25,26^. Therefore, the A domain has a fundamental impact on the type of NRPs synthesized by NRPS and its function during pathogenicity^27^. Adenylation domain is responsible for recognition, activation and incorporation of an amino acid onto the T domain or PCP with high conserved serine which carries a 4′-phosphopantetheine [Ppant] cofactor and a thioester bond is made. The Ppant arms have to penetrate from opposite sides of the C domain to reach the conserved active-site motif HHxxxDG. The second histidine in this motif has been suggested to work as the general base to promote a nucleophilic attack of the a-amino group^28^. C domain catalyzes a condensation reaction to create peptide bonds in non-ribosomal peptide biosynthesis. This domain is located on the carboxyl side of a pp-binding domain. Therefore, C domain plays an essential role in the formation and elongation of the peptide chain in NRPSs^29^.

Three classifications are proposed for the known NRPS biosynthesis: linear (type A), iterative (type B) and non-linear (type C). Linear type as in ACV synthetase, peptaibol synthetases, and cyclosporin synthetases was identified. In an iterative type such as enniatin synthesis, the modules and domains are repeated during the building of a compound. The construction of non-linear NRPSs is more complex and varies from those of linear type. These unusual structures are unpredictable and found in many bacterial NRPSs while most fungal NRPSs are described as linear and iterative types^12^.

Several tools such as, ClustScan^30^, CLUSEAN^31^, SBSPKS^32^, SMURF^33^, PRISM^34^, SeMPI v2^35^ and antiSMASH (Antibiotics and secondary metabolite analysis shell)^36^ have been developed to identify and analyze the enzymatic domains in multi-modular PKSs and/or NRPSs which are the essential enzymes for the production of clinically crucial secondary metabolites. These advanced tools predict the feature of substrates which have vital roles in biosynthetic steps and the chemical structure of the final bioactive compounds based on the genome sequence^36^.

The objectives of this study were (1) to isolate, identify and characterize NRPS gene of *G. boninense*, (2) to determine the relationships among various fungal NRPSs, and (3) to investigate the role of NRPS in the pathogenicity of *G. boninense* during its interaction with oil palm.

## Results

### Identification of NRPS domains

With the aid of antiSMASH and SeMPI v2, contigs containing putative NRPS biosynthesis gene clusters were identified in both *G. boninense* strains NJ3 and G3 whole genome. One putative NRPS biosynthesis gene cluster was detected in both strains. NRPS gene in strain NJ3 was predicted in contig 5393 (Accession Number: LFMK01009681.1), cluster 11 (the location of Cluster 11 was at 1–36,106 bp and the NRPS gene was predicted at 17,490–31,700 bp region in this cluster) via antiSMASH, while the SeMPI v2 demonstrated several genes relevant to A domain. NRPS gene in strain G3 was detected in backbone_133 (Acc. No: PJEW02000037.1), Cluster 4, from 76,088 to 130,317 bp and the NRPS region was defined at 96,088–110,317 bp by antiSMASH. On the other hand, the result obtained from the analysis of SeMPI v2 revealed multiple NRPS genes in G3 strains. All predicted NRPS regions for both NJ3 and G3 strains by antiSMASH and SeMPI v2 were analyzed using BLASTx to compare similarity with NRPS genes of other fungi. The confirmation of NRPS gene requires the presence of A, T and C domains as well as in a single continuous sequence^11^. BLASTx results based on SeMPI v2 showed three regions related to NRPS domains in strains NJ3 and G3 that belong to contigs with Acc. No: LFMK01009681.1 and PJEW02000037.1, respectively. The regions of prediction included 28,767–28,974, 29,403–29,739, and 29,739–30,843 in strain NJ3, and 107,383–107,590, 108,019–108,355, and 109,261–109,459 in strain G3 (See Supplementary Tables S1 and S2). This finding was consistent with BLASTx results that were obtained by antiSMASH, therefore, in both prediction methods, the results showed both NJ3 and G3 had one NRPS gene that possessed three conserved domains and had high similarity with NRPS region of *G. sinense* (Acc. No: PIL24012.1) (for more details refer to Supplementary Tables S3, S4, S5 and S6).

Since there was a continuity in the NRPS sequences by the antiSMASH results and the predicted NRPS regions by SeMPI v2 were located in these ranges, the NRPS regions were selected based on the antiSMASH result for the next steps and the nucleotide locations of different domains [A-C-T] were characterized by using NCBI's Conserved Domain Database (CDD) in each strain. The length of NRPS gene in the related cluster in both strains was about 14,000 bp and NRPS domains (A-T-C) region started from 6000 bp and continued till 14,000 bp.

### Confirmation of NRPS domains

**Figure 1:**
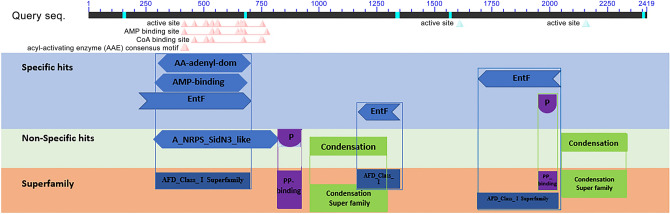
Conserved domains on predicted protein sequence using NCBI Conserved Domain Search^65^. Protein classification: AMP-binding and AA-adenyl-domain belong to adenylation domain, A_NRPS_SidN3_Like is the adenylation A domain of siderophore-synthesizing NRPS^37^, EntF is a NRPS synthetase component F that is responsible for secondary metabolites biosynthesis, catabolism and transport^85^. PP_binding (phosphopantetheine attachment site) is a 4′-phosphopantetheine prosthetic group is linked by a serine. The role of this prosthetic group as a ‘swinging arm’ for the connection of activated fatty acid and amino-acid groups to Condensation domain^29^. Condensation catalyzes a condensation process to form peptide bonds in NRP biosynthesis^86^. AFD_Class_I superfamily, Condensation superfamily, pp-binding superfamily.

Seventeen PCR amplicons were sent for nucleotide sequencing. In this analysis, the length of all amplicons was corresponding to the predicted size. Overall, the length of 8322 bp nucleotides was detected using PCR as NRPS region (*GBNRPS*) in strain UPMGB001 and 2419 aa putative protein were predicted using AUGUSTUS software. The detected DNA region of NRPS in the UPMGB001 strain was aligned with both *G. boninense* strains NJ3 and G3 for more confidence (more details were included in Supplementary Tables S7 and S8). AUGUSTUS prediction, *GBNRPS* contains 14 intron regions. The nucleotide sequence of *GBNRPS* is available in NCBI with accession number MT675190. NCBI’s CDD- search found super-families associated with the NRPS region including AFD_class_I, AMP-binding, Condensation and pp-binding (Fig. 1). Moreover, A_NRPS_SidN3_like represented in Fig. 1, has been identified as the specific structure in the A domain of NRPS (the third adenylation domain of NRPS SidN) that led to making a specific amino acid that belonged to siderophore-synthesizing NRPS^37^.

### Structural analyses of NRPS domains

**Figure 2:**
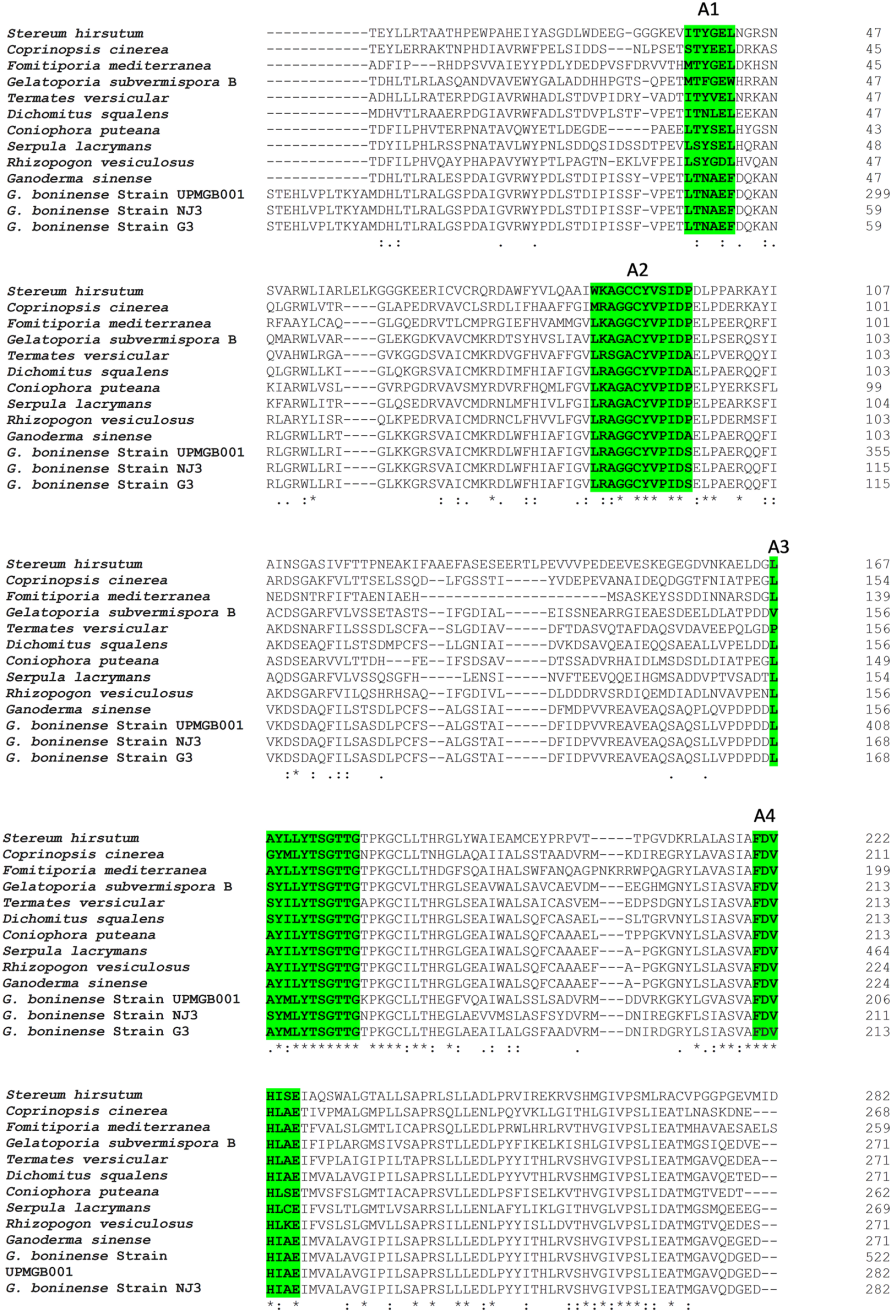

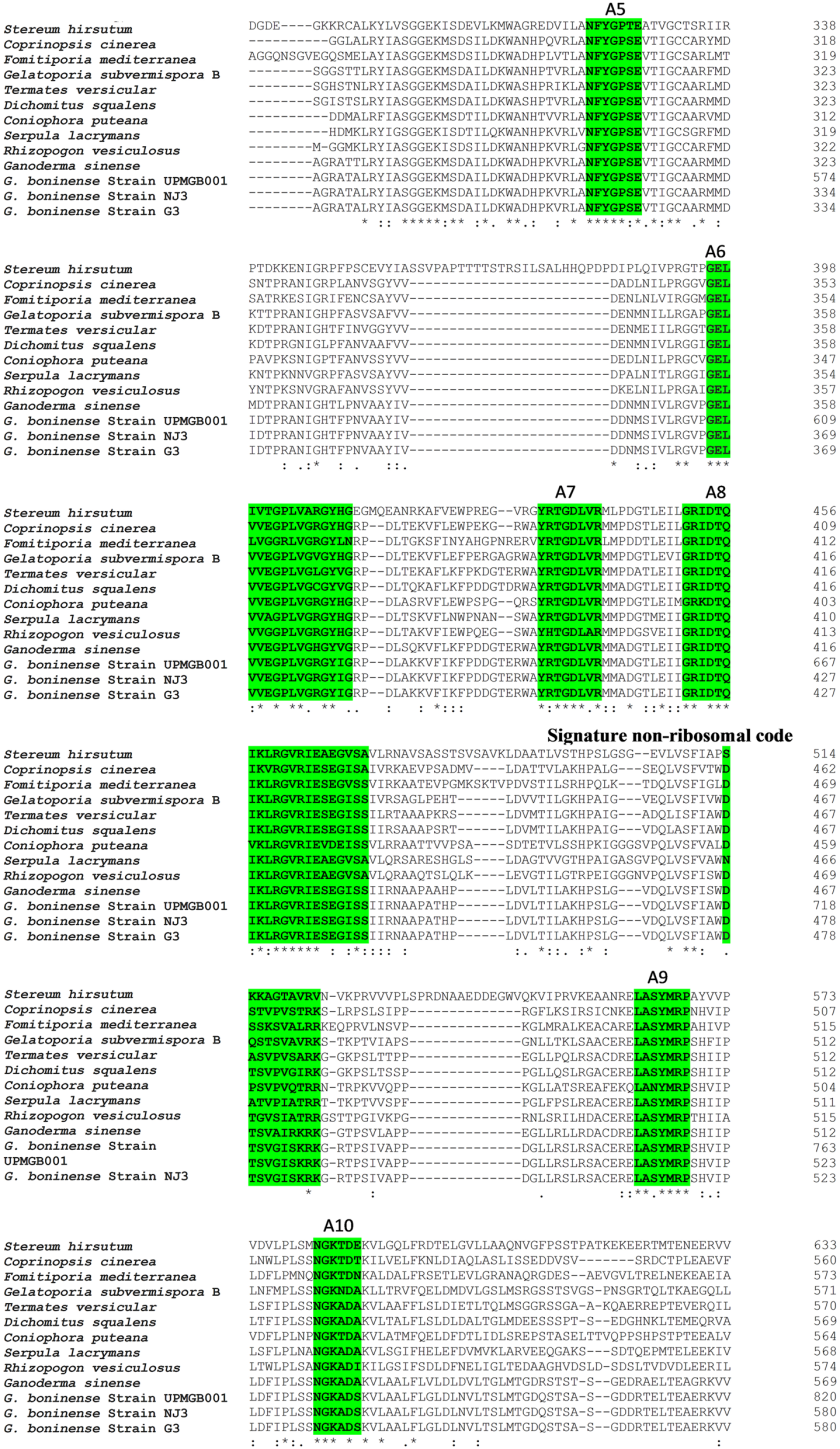

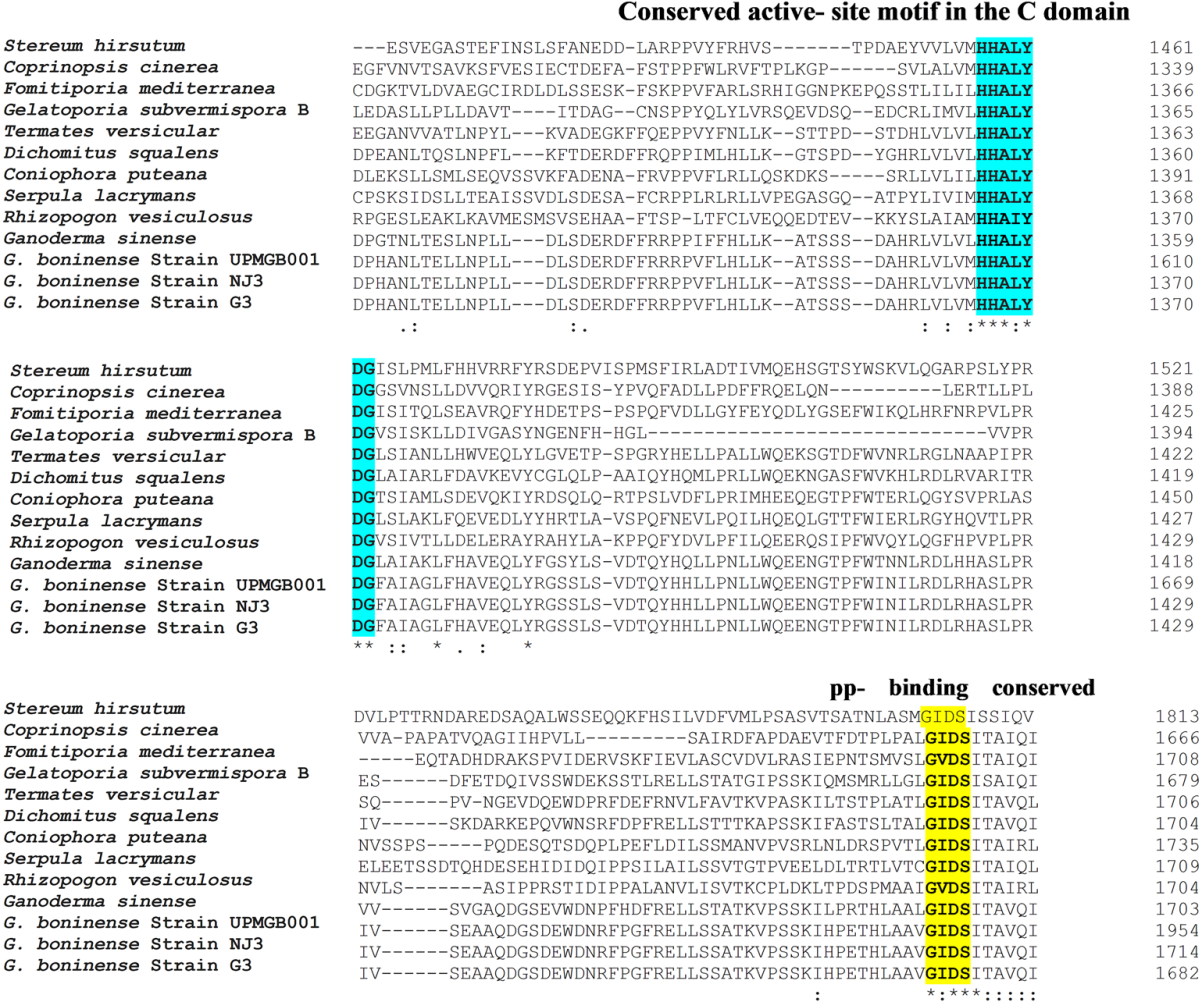
Multiple sequence alignment among predicted amino acid sequences of *G. boninense* and some basidiomycete fungi including: *Stereum hirsutum* (XP_007301650), *Coprinopsis cinerea* okayama7#130 (XP_001833231.2), *Fomitiporia mediterranea* (XP_007263924.1), *Gelatoporia subvermispora* B (EMD38714.1), *Termates versicular* (XP_008036091.1), *Dichomitus squalens* LYAD-421 SS1 (XP_007361133.1), *Coniophora puteana* (XP_007764714.1), *Serpula lacryman****s*** var. lacrymans S7.9 (XP_007324654.1), *Rhizopogon vesiculosus* (OJA08148.1), *Ganoderma sinense* (PIL24012.1), *G. boninense* strain UPMGB001,*G. boninense* strain NJ3 and *G. boninense* strain G*3*. Consensus key: *—fully conserved residue. **:**—conservation of strong groups, **.**—conservation of weak groups, – no consensus. Green box: the conserved Motif Core of A1–A10 and signature non-ribosomal code in A domain. Light blue box: conserved active-site motif in C domain. Yellow box: conserved domains of pp-binding.

Multiple sequence alignment (MSA) among NRPS protein sequences of some basidiomycetes and strain UPMGB001 represented conserved motifs of three main NRPS domains (as shown in Fig. 2). A set of conserved sequence motifs (A1-A10) was detected for A domains according to codes reported by Eisfeld (2009) in Table 1. The side-chain of signature non-ribosomal code of SidNA3 (DXXXXXXXXK) was recognized in the A domain of basidiomycetes^25,38^; DTSVGISKRK sequence code was detected in UPMGB001 strain. UPMGB001 presented a similar conserved region in A1 (conserved motif: LTNAEF), A2 (LRAGLLLVPID), A3 (LAYILYTSGTTGTPKGC), A4 (FDVHIAE), A5 (NFYGPSE), A6 (GELVVEGPLVGRGYIG), A7 (YRTGDLVR), A8 (GRIDTQIKLRGVRIESEGISS), A9 (LASYMRP) and A10 (NGKADA) with some variances in conserved motif codes (as demonstrated in Fig. 2). The core motif of T domain in NRPSs is GXXS^25,26^*. G. boninense* showed a conserved domain GIDS in the region of PP-binding (Fig. 2). Ppant arms have to penetrate from opposite sides of the C domain to reach the conserved active-site motif HHxxxDG^25,28^. HHALYDG as a conserved motif of the C domain region was found in our strain (Fig. 2).

**Table 1:** Conserved A domain motifs of NRPS protein sequences in bacteria, ascomycetes and basidiomycetes^12^ compared with* G.* boninense.

Fungi name	A-domain core motifs									
	A1	A2	A3	A4	A5	A6	A7	A8	A9	A10
Bacteria^87^	L(TS)YxEL	LKAGxAYL(VL)P(LI]D	LAYxxYTSG(ST)TGxPGK	FFDxS	NxYGPTE	GELxIxGxG(VL)ARGYL	Y(RK)TGDL	GRxDxQVKIRGxRIELGEIE	LPxYM (IV)P	NGK(VL)DR
*Cochliobolus heterostrophus* (ascomycete)^56^	LTYxEL	LKAGA (AG)F(VY]P(LI)DP	LAY(VI)(IL)FTSGTGxPKGV	FFDxSxxE	N(GA)YGP(TA)E	GELxIxxxGxG (VL)ARGYL	RxY(RK)TGDLxR	GRxDxQVK(IL)RGxR(IV)ELGEIE	LPxxMxP x	SGKxDR
*Omphalotus olearius *(basidiomycete)^12^	xTYxxL	LKAGxAxxPxx	xAYxxxTSG(TS)TGxPKxV	FFDxxxx (ED)	NxYGPxE	GELx(IVL)GGxxx(AG)xGYxx	X(YF)xTGDxxR	GRxDxxxVKxxGxR (VI)xLxE (IV)x	LPxxMxP	xGKxDR
*Ganoderma boninense* strain UPMGB001	LTNAEF	LRAGGCYVPID	LAYILYTSGTTGTPKG	AFDVHIAE	NFYGPSE	GELVVEGPLVGRGY	YRTGDLVR	GRIDTQIKLRGVRIE	LASYMRP	NGKADS
*G. boninense* strain NJ3	LTNAEF	LRAGGCYVPID	LAYILYTSGTTGTPKG	AFDVHIAE	NFYGPSE	GELVVEGPLVGRGY	YRTGDLVR	GRIDTQIKLRGVRIE	LASYMRP	NGKADS
*G. boninense* strain G3	LTNAEF	LRAGGCYVPID	LAYILYTSGTTGTPKG	AFDVHIAE	NFYGPSE	GELVVEGPLVGRGY	YRTGDLVR	GRIDTQIKLRGVRIE	LASYMRP	NGKADS

**Figure 3:**
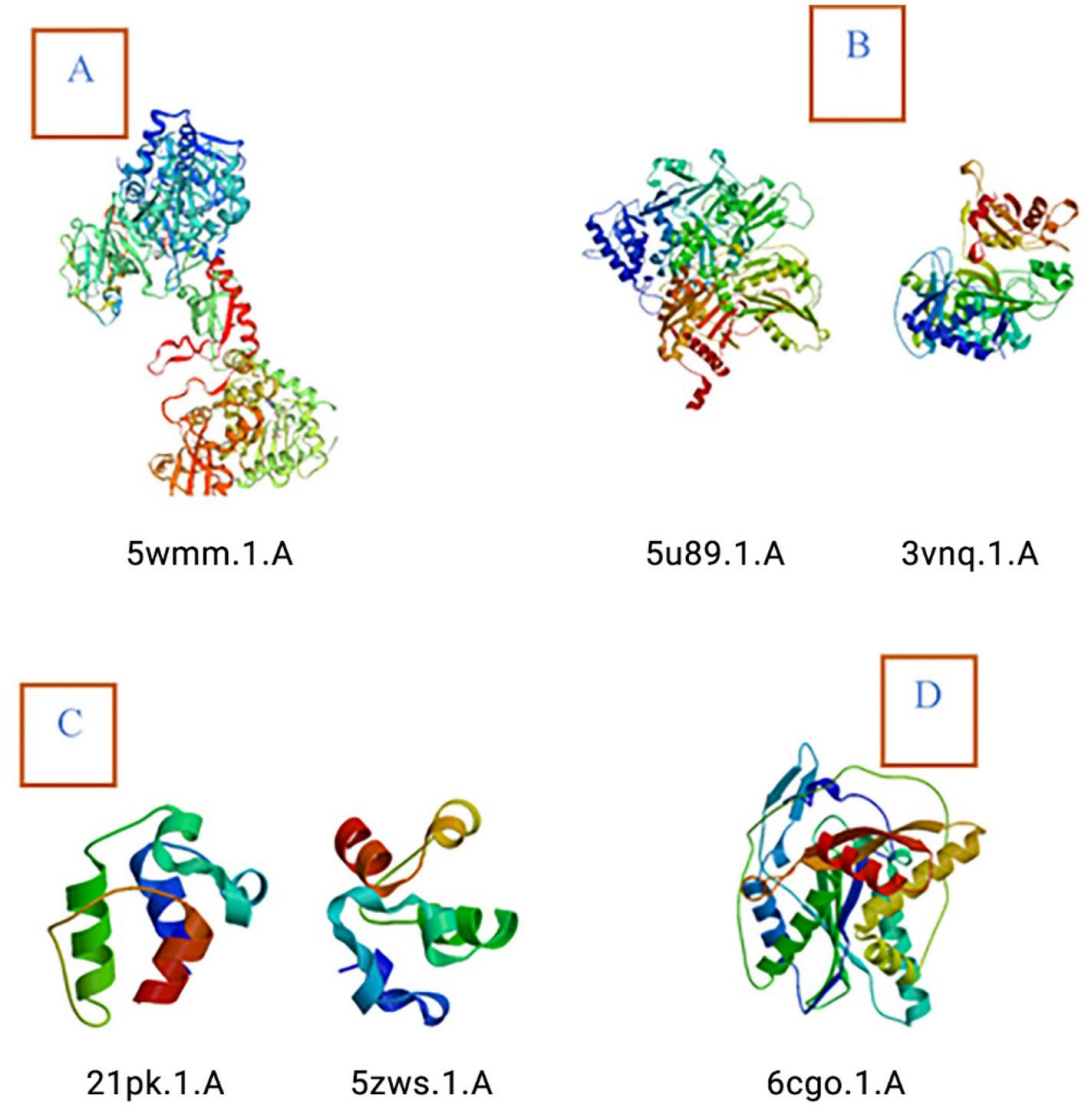
SWISS-MODEL; 3-D NRPS sequence and NRPS domain fragments using templates: (**A**) 5wmm.1.A (PDB code for NRPS). (**B**) 5u89.1.A (PDB code for amino acid adenylation domain), 3vnq.1.A (PDB code for NRPS adenylation protein CytC1). (**C**) 2lpk.1.A and 5zws.1.A (PDB Specialized acyl carrier protein and Acyl carrier protein for PCP domain]. (**D**) 6cgo.1.A (PDB code C domain).

Based on BLASTp result, NRPS of *G. boninense* were found to be homologous to *G. sinense* (Acc. No: PIL24012.1; 85% similarity) and other basidiomycetes such as *Dichomitus squalens* (Acc. No: XP_007361133.1; 66% similarity), *Polyporus arcularius* (Acc. No: TFK91049.1; 61% similarity) and ascomycetes fungi such as *Aspergillus* sp (30% similarity). Secondary structure analyses revealed that NRPS of *G. boninense* strain UPMGB001 consisted of 41.63% alpha-helix, 13.97% beta-sheet, 4.13% beta-turn, and 40.26% random coil. All the predicted models overlapped with their templates showing alpha helices and beta sheets and gave a conformational pattern similar to that of the known NRPSs. Also, ligand ATP which is important for the catalytic activity of non-ribosomal peptide synthetases^39^ was detected in connection with chain A in the Three-dimensional (3-D) model of UPMGB001 NRPS using template 3vnq.1.A (PDB code for NRPS adenylation protein CytC1) (Fig. 3). Moreover, a Phyre2 homology model indicated close structural similarity with 32% sequence identity to SidN-siderophore synthetase module of NRPS.

### Phylogenetic analysis

**Figure 4:**
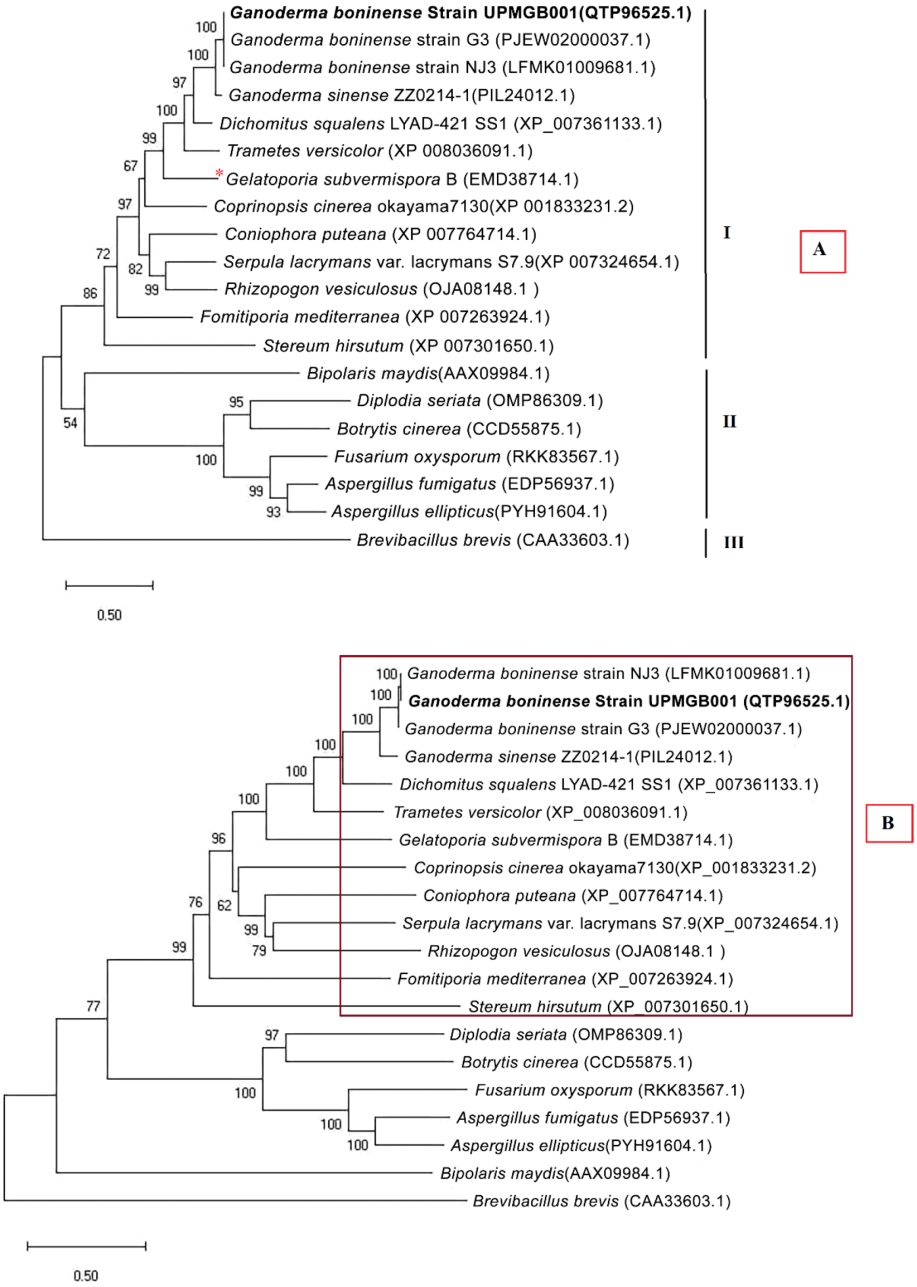
(**A**) Neighbour-joining tree of NRPS peptide sequence. Organism names and accession numbers are indicated on leaves. Bootstrap values upper 50% are demonstrated above the branches. The scale bar shows the number of amino acid replacements per site. I: Basidiomycete' fungi, II: Ascomycete' fungi. NRPS protein sequences in basidiomycetes and ascomycetes were selected based on BLASTp results. III: *Brevibacillus brevis* (Acc No: CAA33603.1) was defined as an outgroup^40,76^. (**B**) Phylogenetic tree evaluating the position of A domain sequences of NRPS protein region in *G. boninense*. Similarity between phylogenetic trees reported by Brandenburger et al.^40^ and the results of this study using the same sequences showed Ganoderma species belong to class VI clade. In their study, Organism and accession numbers belong to the monophyletic type VI clade, a subclade of N5-acyl-N5-hydroxy-l-ornithine [acyl-HO]-activating A domains and particularly of basidiomycete sequences were indicated. *Gelatoporia subvermispora = *Ceriporiopsis subvermispora.*

In this study, we performed the phylogenetic analysis of A domain that has the highest conserved domain among the other main domains (T and C domains)^11^, and also all domains of NRPS with the intent to deduce concordance between them. As presented in Fig. 4, bootstrap values at the inner nodes did not show any significant variations. Comparison between the phylogenetic dendrogram reported by Brandenburger et al.^40^ and the results of this study using the same sequences showed *G. boninense* is placed in the same clade in both studies (Fig. 4B).

### Assessment of disease severity

**Figure 5:**
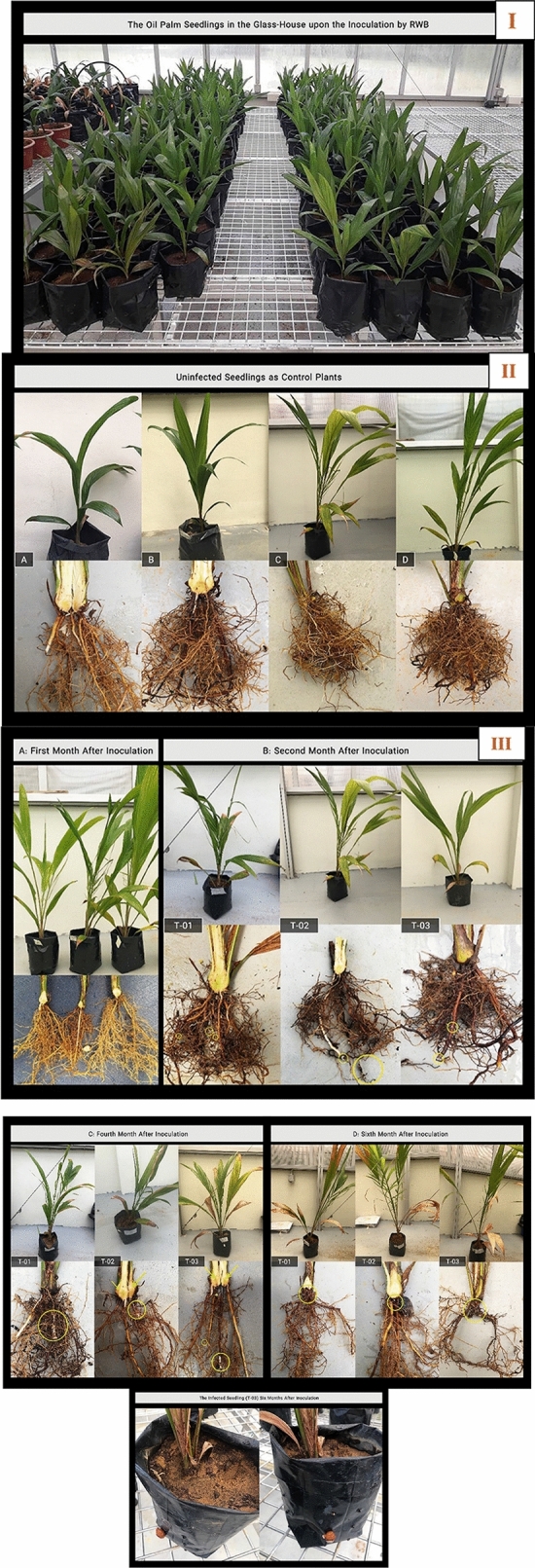
(**I**) All 6-month-old oil palm seedlings (after 1-month adaptation in the greenhouse), 1 h after inoculation by rubber wood Block (RWB); including control (un-inoculated) and infected plants for gene expression and disease severity studies. (**I**) Control (uninfected) seedlings at 1, 2, 4, 6 months after inoculation (MAI); (**A**) control seedling at 1 MAI, (**B**) control seedling at 2 MAI, (**C**) control seedling at 4 MAI, (**D**) control seedling at 6 MAI. (**III**) (**A**) Three infected treatments (T-01, T-02, T-03) at 1 MAI. (**B**) Three inoculated treatments (T-01, T-02, T-03) at 2 MAI with one yellowing leave in T-02 and T-03 and the appearance of the white pile of mycelia on several parts of root in all treatments (as shown by yellow circles), (**C**) infected treatments (T-01,T-02, T-03) at 4 MAI with at least two yellowing leaves and one browning leaves and also, growing of the mycelia mass in the roots, (**D**) infected treatments (T-01,T-02, T-03) at 6 MAI with more than three chlorotic leaves, boles of the infected seedlings demonstrated the brown and black coloration; fruiting body is visible in T-03. Yellow circle indicates the mycelia in each treatment and yellow arrow in part (**C**) represents necrosis bole tissue.

**Figure 6:**
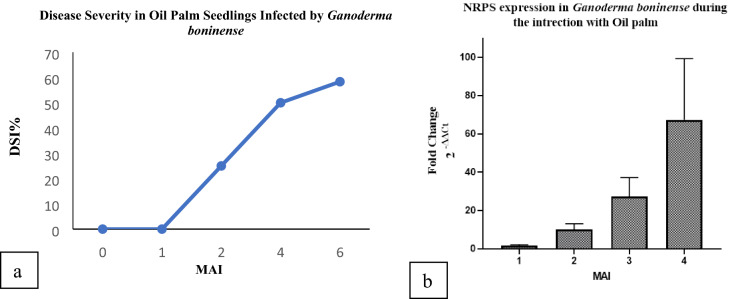
(**a**) Assessment of Disease Severity Index (DSI) of oil palm seedlings challenged with *Ganoderma boninense* at monthly intervals 0, 1, 2, 4, 6 after inoculation based on external disease symptoms and rated based on 0–4 disease severity value^1^. (**b**) Relative quantification of NRPS transcript as fold change (2^-ΔΔct^) during *G. boninense*-oil palm interaction. Bars represent the average mean ± SEM of three technical replicates from three biological replicates. *MAI* month after pathogen inoculation.

All the oil palm seedlings were found to be successfully infected by *G. boninense* with the gradual changes in the appearance of leaves toward necrotic and growth of white mycelial at the base of stems 2 months after artificial inoculation. The un-inoculated plants (Control) did not show any sign of disease symptoms. Observation of symptoms in the infected oil palms by *G. boninense* was examined at every sampling interval [0, 1, 2, 4, 6 months after inoculation (MAI)] according to class disease based on a disease index value of 0–4 as represented in Fig. 5. The infected seedlings were observed with disease severity index (DSI) (%)^1^. The inoculated seedlings showed infection symptoms from 2 MAI (25%) and increased to 50% at 4 MAI. Finally, the inoculated seedlings demonstrated the highest disease severity at 6 MAI with 58.5% as illustrated in Fig. 6a.

### NRPS gene expression in *G. boninense*

The gene expression patterns of NRPS were profiled in *G. boninense* from challenged root tissues of oil palm seedlings at 1, 2, 3 and 4 MAI. These intervals were selected as this time frame was crucial for infection to occur and therefore, was able to determine the involvement of NRPS in the infection process^41^. The efficiency was 93% as measured by LingReg- PCR software. The NRPS transcripts showed 1.54-fold change at 1 MAI, 10.1-fold change at 2 MAI, while NRPS expression was enhanced drastically to 27.33-fold change at 3 MAI. The NRPS transcripts showed the highest abundance with 67.37-fold change at 4 MAI (Fig. 6b).

## Discussion

NRPSs are responsible for producing secondary metabolites and their roles had been identified as virulent factors in different species of ascomycete and basidiomycete fungi^12^. The validated genomic data of *G. boninense* available in NCBI helped to identify the cluster of NRPS using antiSMASH and SeMPI v2 programs and led to the detection of one genomic sequence that is potentially responsible for the production of NRP. This finding is similar to the prediction of only one putative NRPS cluster in *G. sinense* using antiSMASH which had a similar structure to that obtained in *G. lucidum*^42^. The amplification of *G. boninense* NRPS gene (8322 bp nucleotides) revealed the detection of three major superfamilies including AFD_class_I, phosphopantetheine attachment site (pp-binding) and C domain (Fig. 1). However, there were some variations in the DNA sequences among the three strains, NJ3, G3 and UPMGB001 (Supplementary file Table S7) but all the conserved motif domains (A, T and C) have remained stable among them. These conserved motifs in these three strains have the highest similarity compared to other basidiomycetes (Fig. 2, Table 1).

The comparison of NRPS conserved motifs in basidiomycete fungi displays much more variations than those of bacterial origin, even within the same species^12^. However, mutation among species is expected, all ten A motifs in *G. boninense* were detected with minor variances in conserved motif codes. The low sequence similarity of the A domain motif in *G. boninense* compared to that of other basidiomycete fungi could suggest its novelty as indicated in an earlier study on the partial cDNA of 853 nucleotides encoding NRPS from *G. boninense* where core motifs A2, A3 and A5 were detected and showed some variations from other species^43^. As illustrated in Fig. 2 and Table 1, the most diverse variations among A motifs based on the nonribosomal code of conserved motifs for basidiomycetes belong to A1 ((I/L/S/M)(T/S)(Y/F/N)(G/E/V/S/L/A)(E/D)(L/W/F)), and the code LTNAEF have been identified similarly in *Ganoderma* species in this study. Nevertheless, there was a small variation in A2, A3 and A10 between *G. sinense* and *G. boninense*. Interestingly, a minor diversity in A3 showed among UPMGB001 and G3 (L**A**YMLYTSGTTG) with NJ3 (L**S**YMLYTSGTTG) that demonstrated some mutations in the same species among different strains. Most variations in A6 motif belong to *Fomitiporia mediterranea* (GEL**L**V**G**G**R**LVGRGY**LN**). The highest similarity among A motifs belongs to A5, A7 and A9 with NFYGPSE, YRTGDLVR and LASYMRP codes, respectively. While *Stereum hirsutum* has a variance in only one amino acid (NFYGP**T**E**)** in A5, *Rhizopogon vesiculosus* has two distinct amino acids in A7 motif code (Y**H**TGDL**A**R), and *Coniophora puteana* has a single variation in the third amino acid in A9 motif code (LA**N**YMRP). On the contrary, A8 code (GRIDTQ(I/V) K(L/V) RGVRIE(S/A/V) (E/D) (V/I) S(S/A)) revealed high similarity among basidiomycetes in this study. Under this evaluation, the order of similarity of core A code in *G. boninense* with other basidiomycete fungi from highest to lowest includes A9 and A5 > A7 > A8 > A6 > A4 > A3 > A10 > A2 > A1.

Based on findings in this study, the core motif of T domain was detected in *G. boninense* (Fig. 1). This core motif includes extremely conserved serine residue that the hydroxyl group operates as an attachment site for Ppant^26,29^. The MSA among UPMGB001 and other basidiomycetes showed a conserved domain GIDS, excluding *R. vesiculosus and F. mediterranea* with a difference in the second amino acid in this conserved region code (G**V**DS). As illustrated in Fig. 2, C domain with a conserved active-site motif was another domain found in this study; the conserved motif code of the C domain detected with HHALYDG sequences in all, except *R. vesiculosus* with HHA**I**YDG motif.

Extensive studies have been conducted in fungal siderophore biosynthesis of *Cochliobolus*, *Fusarium*, *Aspergillus*, and other genera of ascomycetes^44,45^. Besides, siderophores are secondary metabolites produced by NRPSs that are involved in iron uptake. The remarkable role of siderophores was proven in host invasion. They have, potentially, dual functions: they act not only as pathogenesis effectors but defense elicitors in host infection processes. These two functions are also recognized in some protein effectors released by fungal and bacterial pathogens^19^. The role of siderophore in the pathogenicity process by *G. boninense* in oil palm is unknown. Based on previous studies, siderophores synthesized by NRPS genes are crucial effectors to suppress the immune mechanism of the host plant^19^. A large number of investigations have revealed that lack of extracellular siderophores has a prominent impact on the reduction of the pathogenicity against the host^46^. The release of siderophores as toxic effectors in pathogenic fungi led to inhibiting ROS in the host attacked^47^. Detoxification of ROS is an essential function in the infection process via pathogen, such as the infection of citrus by *A. alternata*^16^. Studies on *F. gramineasum*, *A. brassiciola*, *C. miyabeanus* had demonstrated siderophores played a crucial role in the pathogenicity of wheat, *Arabidopsis*, and rice, respectively^48^. Consequently, a better perception of the mechanism through which siderophore impacts the oil palm immunity, and identification of interactive proteins from oil palm may contribute to the development of new oil palm genotypes resistant against *G. boninense*.

Siderophore producing NRPSs dependent on the domain structure are classified into types I–VI^49^. Diverse enzymes act as catalysts leading to siderophore synthesis with monomeric substrates. These enzymes are monooxygenase-mediated in two steps which consist of hydroxylation and acylation of the N-5 nitrogen atom of l-ornithine via an acyltransferase. In contrast to ascomycetes, information about siderophore production by basidiomycetes are widely unrecognized including genetic and enzymatic necessities for siderophore production. Only two reports on NRPS-related genes in basidiomycetes were found; ferrichrome and ferrichrome A biosynthesis in *Ustilago maydis*^50^, and ferrichrome A biosynthesis genes in *Omphalotus olearius*^51^.

According to prior studies, the specific putative NRPS gene (*CsNPS2*) in the genome of white-rot model species *Ceriporiopsis subvermispora* (or *Gelatoporia subvermispora*) is a member of an extensively distributed but formerly unknown class (type VI) of fungal siderophore synthetases^40^. A study on the type of siderophore-producing NRPSs in *C. subvermispora* as a white-rot basidiomycete has shown that it can be a model for a myriad of type VI basidiomycete NRPSs^40^. Furthermore, the similarity of the this study with that of Brandenburger et al.^40^ confirmed NRPS gene in *G. boninense* belongs to the type VI siderophore family. Phylogenic analysis displayed this type of siderophore belongs to the iterative NRPS template^40^. Specificity-conferring residues of SidN3 (SidNA3) of fungi were used in the phylogenic analysis. *SidN* gene is responsible for encoding the typical siderophore type, known as epichloenin. Based on the studies to date, SidNA3 has been detected only in the eukaryotic NRPS domain^52,53^. Although there is not enough information about the structure of siderophore in *G. boninense*, SidNA3 was detected in NRPS sequence domain in UPMGB001 strain (Figs. 1, 2). There are some reports about siderophore production in *Ganoderma* sp. For instance, siderophore synthesis has been studied in *G*. *boninense* by Azurol S (CAS) agar plate assay^43^, siderophores produced by *Ganoderma* sp. was screened using both culture supernatants and solid medium by the universal CAS agar plate assay and the result showed that siderophore synthesized by *Ganoderma* sp. is under the hydroxymate type^54^ (there are three main classes of siderophores include hydroxamate, catecholate and carboxylates^55^).

*Ganoderma boninense* is the causal agent of BSR disease, however, the precise infection pathway of *G. boninense* is still unraveled^5^. Thus, attempts to identify the pathogenicity genes of *G. boninense* involved in the infection process on oil palm is crucial. NRPS gene was expressed as early as 1 MAI^56^, as demonstrated in our study. Lee et al.^56^ studied the role of NRPS at the infection stage of *C. heterostrophus* during the plant-pathogen interaction on maize roots. Twelve NRPSs in the genome of this fungus have been detected, and their roles as virulent factors were investigated. Each of the NRPSs was eliminated one after another, and only NPS6 has shown to be a virulent factor to maize. The removal of partial or complete NPS6 caused a reduction in virulence and an increase in H_2_O_2_ sensitivity^56^. Additionally, more investigations highlighted the role of extracellular siderophore synthesis in the expression of cell wall synthesis enzymes, whilst the loss of *sid1* remarkably reduced the expression of genes encoding the enzymes that are responsible for cell wall synthesis. Appressoria and conidia in *C. heterostrophus* have a significant role in the penetration of host cell that are synthesized by *sid1* and *NPS6*, therefore, deletion of them strictly disturbed the pathogenicity ability in the fungus^46^. Comprehensive research on 22 NRPS genes of *T. virens* showed seven of them upregulated when the mycelia of the fungus were in contact with the roots of maize, and they caused internal colonization in the root during the infection on the host^57^. Investigation on differential expression of 437 genes of *Valsa mail*, as a destructive pathogen of apple in China, revealed the expression of NRPS transcript occurred more than 100 times during the fungal infection and that NRPS was involved with the production of toxic SM during the pathogenicity in apple^58^.

The relative fold-changes of expression in different months of root tissue colonization were calculated by comparing the expressions of NRPS in the C domain (identified in this study) and the previous study on the A domain (identified by Wong et al.^43^); the results showed the same trend in both studies. Consistent with our investigation in Fig. 6, NRPS gene expression showed an outstanding increase of 67 change at 4 MAI when DSI of infected seedlings was relatively high (50%). Infection of the oil palm roots by *G. boninense* causing basal stem tissue rot appeared as typical symptoms during 5 months of observation^59^ as the highest DSI was observed at 6 MAI in this study (58.5%) and Wong et al. (62%). Wong and colleagues studied the expression level of NRPS A domain in *G. boninense* strain PER71 using reverse transcriptase-PCR. As a result, NRPS was detected at 1–4 MAI when no apparent disease symptoms observed in infected oil palm seedlings, while disease symptoms appeared at 4 MAI with 45% DSI and reached a peak at 6 MAI with 62% DSI^43^.

The findings of this study enhance our understanding of NRPS gene structure of *G. boninense* and its involvement in the disease progression of BSR as an effector gene. The correlation between high disease severity and high expression of NRPS gene indicated that this gene in *G. boninense* played a significant role in the advancement of BSR disease. Moreover, these results could lead to the development of an early detection system using NRPS region as a *Ganoderma*-specific biomarker for efficient management of BSR disease in oil palm.

## Materials and methods

### Fungal strain and culture conditions

*Ganoderma boninense* strain UPMGB001 was obtained from the Culture Collection Unit, Department of Plant Protection, Faculty of Agriculture, Universiti Putra Malaysia (Permission to use the culture was obtained from the head of department). The culture was kept and maintained on malt extract agar (MEA) (Difco-Oxoid, UK) plates. Seven-day-old culture agar discs (3 mm diameter) were inoculated into 50 ml potato dextrose broth (PDB) (Difco-Oxoid, UK) in 100 ml Erlenmeyer flask. The flasks were covered with aluminium foil, sealed with parafilm tape and left for 14 days before harvesting for genomic DNA (gDNA) isolation.

### Identification of NRPS gene in *Ganoderma boninense*

To identify contigs that might contain NRPS, *G. boninense* strain NJ3 whole-genome shotgun sequence data (Acc. No: LFMK00000000)^60^, and *G. boninense* strain G3 whole-genome shotgun sequence data (Acc. No: PJEW00000000)^61^ were submitted to the fungal antiSMASH software version 4.2.0^38,62^, https://antismash.secondarymetabolites.org/, a web server for forecasting NRPS and PKS along with the screening of metabolites in product databases^35^; and SeMPI v2, http://sempi.pharmazie.uni-freiburg.de/, to analyze fungal genomes for their possible capability of secondary metabolite synthesis^36^. The analysis using antiSMASH was modified by the prediction of open reading frames (ORFs), the ORFs of the NRPS gene was anticipated by AUGUSTUS software, http://bioinf.uni-greifswald.de/webaugustus/^63^, based on *Coprinus cinerea* gene models with the default program parameters. The results of prediction via antiSMASH and SeMPI v2 was initially analyzed by BLASTx searches against GenBank database^64^ for any non-ribosomal peptide synthetase that forms the basis of an NRPS cluster. Identification of NRPS gene was based on similarity to NRPS gene sequence in *G. sinense* annotated in NCBI by accession number PIL24012.1^42^. Characterization of the domains present in these genes was done using NCBI’s CDD^65^. A putative NRPS gene which included at least three main conserved domains (A, T and C) was confirmed for the next steps^11^. The results obtained for G3 and NJ3 strains were also used for phylogenetic analysis.

### Amplification of NRPS domains

The NRPS region identified by antiSMASH and SeMPI v2 was verified by using a PCR-based approach. DNA was extracted from UPMGB001 strain that was grown on a medium containing PDA using Cetyltrimethylammonium bromide (CTAB) DNA Extraction Protocol^66–68^. Seventeen primer sets were designed to target NRPS domains using software Primer3^69^. Details about the design of primers and the list of primers are presented in Supplementary File Table S9. PCR amplification was performed using Dream Taq PCR Master Mix (2X), (Thermo Fisher Scientific, USA). PCR products were analysed using electrophoresis in 1% (w/v) agarose gels stained with FloroSafe DNA (Thermo Fisher Scientific, USA).

### Data analysis

The amplified PCR products were sent to First Base Laboratories Sdn. Bhd., Malaysia, for DNA sequencing. Sanger method and BigDye Terminator v3.1 Cycle Sequencing Kit (Thermo Fisher Scientific, USA) used by the company. Consensus sequences were constructed with BioEdit Sequence Alignment Editor Program; overlapping sequences between consensus sequences were found using Multiple Sequence Alignment (CLUSTAL W)^70^; the errors were checked by BioEdit and were corrected manually, and results were analyzed using BLASTx^71^. Coding sequences and probable protein were predicted with AUGUSTUS software^63^ based on *Coprinus cinerea* gene models. Conserved domains were analyzed using NCBI’s CDD-search, Putative amino acid sequences were analyzed using BLASTp^71^.

### Structure analyses of non-ribosomal peptide synthetase domains

Multiple sequence alignment (MSA) of protein domains of NRPS among UPMGB001 strain and other basidiomycetes was carried out to evaluate the degree of similarity of conserved motifs in different domains in NRPS region using Clustal Omega, https://www.ebi.ac.uk/. Secondary structures were predicted using SOPMA, https://npsa-prabi.ibcp.fr/cgibin/npsa_automat.pl?page=npsa_sopma.html^72^ and homology analyses of the structure were done using Phyre server, http://www.sbg.bio.ic.ac.uk/phyre/^39^. 3-D structure modelling of the polypeptides was done using the automated mode of the SWISS-MODEL tool on ExPASy website, https://swissmodel.expasy.org/^73^. Structure predictions for NRPS in UPMGB001 were made using the following templates: 5wmm.1.A (Protein database (PDB) code for NRPS), 5u89.1.A (PDB code for amino acid adenylation domain), 2lpk.1.A and 5zws.1.A (PDB Specialized acyl carrier protein and Acyl carrier protein for PCP domain) and 6cgo.1.A (PDB code C domain protein).

### Phylogenetic analysis

The alignments and phylogenetic tree were created with MEGA X software^74^ using the in-built Clustal W alignment engine and the neighbour-joining algorithm^75^. NRPS region from *Brevibacillus brevis* (Acc. No: CAA33603.1) was defined as an outgroup^40,76^. The Clustal W alignment was further applied to determine the nonribosomal specificity codes of SidN3 from fungi obtained by BLASTp. This analysis from basidiomycetes and ascomycetes included 20 amino acid sequences. All ambiguous positions were excluded for each sequence pair [pairwise deletion option].

### Plant materials

A total of fifty-four 5-month-old oil palm (*E. guineensis* Jacq., Dura × Pisifera, GH500 series) seedlings were purchased from Sime Darby Seeds and Agricultural Services Sdn. Bhd. (Banting, Malaysia) for both disease assessment and NRPS expression studies. Seedlings were acclimatized for 1 month in the greenhouse before artificial inoculation.

Fungal Inoculum Preparation. Rubber (*Hevea brasiliensis*) woodblocks (RWBs) of 6 cm × 6 cm × 6 cm were purchased from Huat Hing Sdn. Bhd. (Semenyih, Malaysia), and were prepared for artificial inoculation using the direct sitting technique^77^. RWBs were used as substrate carriers to supply nutrients for *G. boninense* under host-free conditions. Seven days old *G. boninense* cultured on MEA plate was cut into small pieces and equally dispersed on the autoclaved RWBs containing 50 mL of MEA added as supplementary nutrition for *G. boninense*. The inoculated RWBs were kept at room temperature (28 °C) for 4 weeks in darkness until full mycelial colonization**.** Fungal inoculum preparation was carried out according to Govender et al*.*^78^*.*

### Disease severity index (DSI)

Disease assessment with destructive sampling was arranged in inoculated and control plants at 0, 1, 2, 4, 6 MAI with completely randomized design (CRD). Fifteen seedlings were used for each group of inoculated and uninoculated plants. Disease progression of the inoculated seedlings was assessed at 0, 1, 2, 4, 6 MAI according to external disease symptoms and rated based on 0–4 disease severity value (the details were summarized in Supplementary Table S10). DSI was calculated using the formula shown below based on the number of plants showing that disease class per treatment^1^.

$${\text{Disease Severity Index (DSI)}} = \frac{{\sum {{\text{(A}} \times {\text{B)}} \times {100}} }}{{\sum {n \times 4} }} \,$$

A = disease class (0, 1, 2, 3, or 4), B = number of plants showing that disease class per treatment, n = total number of plants per treatment.

### Sample preparation for gene expression observation

Seedlings were divided into two parts; twelve Control plants [non-inoculated, RWBs without *G. boninense*] and twelve inoculated (RWBs with *G. boninense*) plants including three independent biological replicates per treatment. Inoculated and control plants were removed from their polybags at 1, 2, 3, 4 MAI and their root tissues were used for gene expression analysis. The harvested root samples were washed with distilled water, snap-freezed with liquid nitrogen and stored at − 80 °C for RNA extraction. The expression of NRPS of *G. boninense* and BSR disease severity in oil palm seedlings were used to observe the probable correlation between the two parameters.

### Total RNA extraction and first-strand cDNA synthesis

Extraction of total RNA from the frozen root tissue samples (n = 3) was done using RNeasy Mini Kit (Qiagen, Germany) following the manufacturer's procedure. The total RNA concentration and quality were measured using NanoDrop ND-1000 UV–Vis Spectrophotometer (Thermo Fisher Scientific, USA) and analyzed by 1% gel electrophoresis. First-strand cDNA was synthesized from DNase I-treated RNA samples using the Omniscript Reverse Transcription Kit (Qiagen, German) according to instructions from the manufacturer. The cDNA was kept in RNAse free water (DEPC water) and stored at − 20 °C.

### Primer design

The conserved sequence of C domain (catcacgcgctgtacgacggg) was located between forward and reverse primers. Gene-specific primer of NRPS in this study was designed on the Condensation coding region of transcript sequence to evaluate NRPS expression. The primers were designed using Primer3 plus program http://www.bioinformatics.nl/ according to the length between 17 and 22 bp, GC content of 35 and 65% and melting temperature between 59 and 63 °C. The amplicon size was designed to range between 120 and 250 bp. Specific primer sequences, as well as eEF2 and α Tubulin as reference genes for *G. boninense*^79^ were listed in the Supplementary File, Table S11.

### Quantitative real-time PCR (qPCR)

Each qPCR reaction was performed following QuantiNova SYBR Green PCR kit manual (Qiagen, German). Three individual technical replicates were performed for each biological replicate at respective sampling intervals and a non-template control was included to monitor cross-contamination. qPCR was performed using Eppendorf Mastercycler Real-time PCR System (Eppendorf, UK). The baseline, the cycle of threshold (C_t_) and PCR efficiency were determined by LingReg- PCR software^80^. Statistical analysis for comparison between expression of the target gene (NRPS) versus the reference genes (eEf2, α Tubulin ) was computed by Pfaffl method [by the fold change equation (2^−∆∆Ct^)]^81^ and Relative Expression Software Tool (REST2009)^82,83^, this software changes C_t_ values into normalized relative expression values by the fold change equation (2^−∆∆Ct^)^84^. The mean ± SEM was evaluated by one-way ANOVA analysis in Graphpad Prism version 8.0.0 software for windows (P < 0.05 showed a significant difference between the infected group and non-infected group at each interval).

### Ethical approval

All local, national or international guidelines and legislation were adhered to in the production of this study.

## Supplementary Information

Supplementary Information 1.

## References

[1] Kwan Y-M, Meon S, Ho C-L, Wong M-Y (2016). Selection of reference genes for quantitative real-time PCR normalization in Ganoderma-infected oil palm (*Elaeis guineensis*) seedlings. Australas. Plant Pathol..

[2] Chong KP, Dayou J, Alexander A (2017). Detection and control of *Ganoderma boninensein* oil palm crop. SpringerBriefs Agric..

[3] Chiew YL, Shimada S (2013). Current state and environmental impact assessment for utilizing oil palm empty fruit bunches for fuel, fiber and fertilizer—a case study of Malaysia. Biomass Bioenerg..

[4] Ishola, F.* et al.* Nigerian Oil Palm Industry as a Sustainable Renewable Energy Resource. In *E3S Web of Conferences* **152**, 02005 (EDP Sciences, 2020).

[5] Paterson RRM (2019). *Ganoderma boninense* disease of oil palm to significantly reduce production after 2050 in Sumatra if projected climate change occurs. Microorganisms.

[6] Hushiarian R, Yusof NA, Dutse SW (2013). Detection and control of *Ganoderma boninense*: Strategies and perspectives. Springerplus.

[7] Siddiqui Y, Surendran A, Paterson RRM, Ali A, Ahmad K (2021). Current strategies and perspectives in detection and control of basal stem rot of oil palm. Saudi J. Biol. Sci..

[8] Perez-Nadales E (2014). Fungal model systems and the elucidation of pathogenicity determinants. Fungal Genet. Biol..

[9] Scharf DH, Heinekamp T, Brakhage AA (2014). Human and plant fungal pathogens: The role of secondary metabolites. PLoS Pathog..

[10] Keller NP (2019). Fungal secondary metabolism: Regulation, function and drug discovery. Nat. Rev. Microbiol..

[11] Sayari M (2019). Distribution and evolution of nonribosomal peptide synthetase gene clusters in the ceratocystidaceae. Genes.

[12] Eisfeld K, Anke T, Weber D (2009). Non-ribosomal peptide synthetases of fungi. Physiology and Genetics.

[13] Schwarzer D, Marahiel MA (2001). Multimodular biocatalysts for natural product assembly. Naturwissenschaften.

[14] Soukup AA, Keller NP, Wiemann P (2016). Enhancing nonribosomal peptide biosynthesis in filamentous fungi. Nonribosomal Peptide and Polyketide Biosynthesis.

[15] Walton, J. Chemistry of the *Amanita *Peptide Toxins. In* The Cyclic Peptide Toxins of Amanita and Other Poisonous Mushrooms* 19–55 (Springer, Cham, 2018).

[16] Hamid S, Wong M-Y, Chai-Ling H, Abdullah S, Wagstaff C (2017). Elicitors and their roles in plant defence against pathogens particularly basidiomycetes. Crop improvement.

[17] Frías M, González C, Brito N (2011). BcSpl1, a cerato-platanin family protein, contributes to *Botrytis cinerea* virulence and elicits the hypersensitive response in the host. New Phytol..

[18] Collemare J, O'Connell R, Lebrun MH (2019). Nonproteinaceous effectors: The terra incognita of plant–fungal interactions. New Phytol..

[19] Aznar A, Dellagi A (2015). New insights into the role of siderophores as triggers of plant immunity: What can we learn from animals?. J. Exp. Bot..

[20] Condon BJ (2013). Comparative genome structure, secondary metabolite, and effector coding capacity across *Cochliobolus pathogens*. PLoS Genet..

[21] Azizi P (2016). Toward understanding of rice innate immunity against *Magnaporthe oryzae*. Crit. Rev. Biotechnol..

[22] Valent B, Khang CH (2010). Recent advances in rice blast effector research. Curr. Opin. Plant Biol..

[23] Kodama M (2019). Evolution of pathogenicity in Alternaria plant pathogens. J. Gen. Plant Pathol..

[24] Meena M, Samal S (2019). Alternaria host-specific (HSTs) toxins: An overview of chemical characterization, target sites, regulation and their toxic effects. Toxicol. Rep..

[25] Süssmuth RD, Mainz A (2017). Nonribosomal peptide synthesis—principles and prospects. Angew. Chem. Int. Ed..

[26] Miller BR, Sundlov JA, Drake EJ, Makin TA, Gulick AM (2014). Analysis of the linker region joining the adenylation and carrier protein domains of the modular nonribosomal peptide synthetases. Prot. Structu. Funct. Bioinform..

[27] Stanišić A, Kries H (2019). Adenylation domains in nonribosomal peptide engineering. ChemBioChem.

[28] Bloudoff K, Schmeing TM (2017). Structural and functional aspects of the nonribosomal peptide synthetase condensation domain superfamily: Discovery, dissection and diversity. Biochim. Biophys. Acta Prot. Proteom..

[29] Kittilä T, Mollo A, Charkoudian LK, Cryle MJ (2016). New structural data reveal the motion of carrier proteins in nonribosomal peptide synthesis. Angew. Chem. Int. Ed..

[30] Starcevic A (2008). ClustScan: An integrated program package for the semi-automatic annotation of modular biosynthetic gene clusters and in silico prediction of novel chemical structures. Nucleic Acids Res..

[31] Weber T (2009). CLUSEAN: A computer-based framework for the automated analysis of bacterial secondary metabolite biosynthetic gene clusters. J. Biotechnol..

[32] Anand S (2010). SBSPKS: Structure based sequence analysis of polyketide synthases. Nucleic Acids Res..

[33] Khaldi N (2010). SMURF: Genomic mapping of fungal secondary metabolite clusters. Fungal Genet. Biol..

[34] Skinnider MA (2020). Comprehensive prediction of secondary metabolite structure and biological activity from microbial genome sequences. Nat. Commun..

[35] Zierep PF, Ceci AT, Dobrusin I, Rockwell-Kollmann SC, Günther S (2021). SeMPI 2.0—a web server for PKS and NRPS predictions combined with metabolite screening in natural product databases. Metabolites.

[36] Blin K (2013). AntiSMASH 2.0—a versatile platform for genome mining of secondary metabolite producers. Nucleic Acids Res..

[37] Lee TV (2010). Structure of a eukaryotic nonribosomal peptide synthetase adenylation domain that activates a large hydroxamate amino acid in siderophore biosynthesis. J. Biol. Chem..

[38] Kudo F, Miyanaga A, Eguchi T (2019). Structural basis of the nonribosomal codes for nonproteinogenic amino acid selective adenylation enzymes in the biosynthesis of natural products. J. Ind. Microbiol. Biotechnol..

[39] Qadri M (2017). An insight into the secondary metabolism of *Muscodor yucatanensis*: Small-molecule epigenetic modifiers induce expression of secondary metabolism-related genes and production of new metabolites in the endophyte. Microb. Ecol..

[40] Brandenburger E (2017). A highly conserved basidiomycete peptide synthetase produces a trimeric hydroxamate siderophore. Appl. Environ. Microbiol..

[41] Parvin W (2020). Phenazine from *Pseudomonas aeruginosa* UPMP3 induced the host resistance in oil palm (*Elaeis guineensis* Jacq.)-Ganoderma boninense pathosystem. Sci. Rep..

[42] Zhu Y (2015). Chromosome-level genome map provides insights into diverse defense mechanisms in the medicinal fungus *Ganoderma sinense*. Sci. Rep..

[43] Wong, M. Y., Jackie, C., Siti, N., Akmar, A., & Abrizah, O. Molecular analyses of nonribosomal peptide synthetase in *Ganoderma boninense* pat. In *PIPOC 2011 International Palm Oil Congress: Palm Oil Fortifying the world *(2011).

[44] Haas H (2014). Fungal siderophore metabolism with a focus on *Aspergillus fumigatus*. Nat. Prod. Rep..

[45] Turgeon BG, Oide S, Bushley K (2008). Creating and screening *Cochliobolus heterostrophus* non-ribosomal peptide synthetase mutants. Mycol. Res..

[46] Oide S, Turgeon BG (2020). Natural roles of nonribosomal peptide metabolites in fungi. Mycoscience.

[47] Chen LH, Lin CH, Chung KR (2013). A nonribosomal peptide synthetase mediates siderophore production and virulence in the citrus fungal pathogen A lternaria alternata. Mol. Plant Pathol..

[48] Fatima N (2017). Siderophore in fungal physiology and virulence. J. Pharmacog. Phytochem..

[49] Bushley KE, Ripoll DR, Turgeon BG (2008). Module evolution and substrate specificity of fungal nonribosomal peptide synthetases involved in siderophore biosynthesis. BMC Evol. Biol..

[50] Winterberg B (2010). Elucidation of the complete ferrichrome A biosynthetic pathway in *Ustilago maydis*. Mol. Microbiol..

[51] Welzel K, Eisfeld K, Antelo L, Anke T, Anke H (2005). Characterization of the ferrichrome A biosynthetic gene cluster in the homobasidiomycete *Omphalotus olearius*. FEMS Microbiol. Lett..

[52] Koulman A (2012). Identification of extracellular siderophores and a related peptide from the endophytic fungus Epichloë festucae in culture and endophyte-infected *Lolium perenne*. Phytochemistry.

[53] Sørensen JL (2014). Fungal NRPS-dependent siderophores: From function to prediction. Biosynthesis and Molecular Genetics of Fungal Secondary Metabolites.

[54] Kumari RS, Kaviyarasan V (2014). Screening of siderophores in basidiomycetes. J. Pharm. Biomed. Sci..

[55] Sahu S, Prakash A (2021). Berberine as siderophore from *Talaromyces trachyspermus*: Augmentation and characterization. bioRxiv.

[56] Lee B-N (2005). Functional analysis of all nonribosomal peptide synthetases in *Cochliobolus heterostrophus* reveals a factor, NPS6, involved in virulence and resistance to oxidative stress. Eukaryot. Cell.

[57] Mukherjee PK, Buensanteai N, Moran-Diez ME, Druzhinina IS, Kenerley CM (2012). Functional analysis of non-ribosomal peptide synthetases (NRPSs) in *Trichoderma virens* reveals a polyketide synthase (PKS)/NRPS hybrid enzyme involved in the induced systemic resistance response in maize. Microbiology.

[58] Ke X (2014). Transcriptome profiling to identify genes involved in pathogenicity of *Valsa *mali on apple tree. Fungal Genet. Biol..

[59] Rees R, Flood J, Hasan Y, Potter U, Cooper RM (2009). Basal stem rot of oil palm (*Elaeis guineensis*); mode of root infection and lower stem invasion by *Ganoderma boninense*. Plant. Pathol..

[60] Mercière M (2015). Identification and development of new polymorphic microsatellite markers using genome assembly for *Ganoderma boninense*, causal agent of oil palm basal stem rot disease. Mycol. Prog..

[61] Utomo C (2018). Draft genome sequence of the phytopathogenic fungus *Ganoderma boninense*, the causal agent of basal stem rot disease on oil palm. Genome Announc..

[62] Blin K (2017). antiSMASH 4.0—improvements in chemistry prediction and gene cluster boundary identification. Nucleic Acids Res..

[63] Stanke M, Diekhans M, Baertsch R, Haussler D (2008). Using native and syntenically mapped cDNA alignments to improve de novo gene finding. Bioinformatics.

[64] Boratyn GM (2012). Domain enhanced lookup time accelerated BLAST. Biol. Direct.

[65] Marchler-Bauer A (2010). CDD: A conserved domain database for the functional annotation of proteins. Nucleic Acids Res..

[66] Cullings K (1992). Design and testing of a plant-specific PCR primer for ecological and evolutionary studies. Mol. Ecol..

[67] Doyle J, Doyle J (1987). CTAB DNA extraction in plants. Phytochem. Bull..

[68] Clarke JD (2009). Cetyltrimethyl ammonium bromide (CTAB) DNA miniprep for plant DNA isolation. Cold Spring Harbor Protoc..

[69] Untergasser A (2012). Primer3—new capabilities and interfaces. Nucleic Acids Res..

[70] Larkin MA (2007). Clustal W and clustal X version 2.0. Bioinformatics.

[71] Madden T (2013). The BLAST sequence analysis tool. The NCBI Handbook [Internet].

[72] Geourjon C, Deleage G (1995). SOPMA: Significant improvements in protein secondary structure prediction by consensus prediction from multiple alignments. Bioinformatics.

[73] Arnold K, Bordoli L, Kopp J, Schwede T (2006). The SWISS-MODEL workspace: A web-based environment for protein structure homology modelling. Bioinformatics.

[74] Kumar S, Stecher G, Li M, Knyaz C, Tamura K (2018). MEGA X: Molecular evolutionary genetics analysis across computing platforms. Mol. Biol. Evol..

[75] Saitou N, Nei M (1987). The neighbor-joining method: A new method for reconstructing phylogenetic trees. Mol. Biol. Evol..

[76] Conti E, Stachelhaus T, Marahiel MA, Brick P (1997). Structural basis for the activation of phenylalanine in the non-ribosomal biosynthesis of gramicidin S. EMBO J..

[77] Kwan Y-M, Meon S, Ho C-L, Wong M-Y (2015). Cloning of nitric oxide associated 1 (NOA1) transcript from oil palm (*Elaeis guineensis*) and its expression during Ganoderma infection. J. Plant Physiol..

[78] Govender NT, Mahmood M, Seman IA, Wong M-Y (2017). The phenylpropanoid pathway and lignin in defense against Ganoderma boninense colonized root tissues in oil palm (*Elaeis guineensis* Jacq.). Front. Plant Sci..

[79] Lim F-H (2014). Isolation and selection of reference genes for *Ganoderma boninense* gene expression study using quantitative real-time PCR (qPCR). J. Oil Palm Res..

[80] Ruijter, J., Van Der Velden, S. & Ilgun, A. Amplification efficiency: linking baseline and bias in the analysis of quantitative PCR data.* Nucleic Acids Res.* **37**, e 45 (2009). 10.1093/nar/gkp045 PMC2665230 19237396

[81] Pfaffl MW (2004). Quantification strategies in real-time PCR. AZ Quant. PCR.

[82] Pfaffl MW, Horgan GW, Dempfle L (2002). Relative expression software tool (REST©) for group-wise comparison and statistical analysis of relative expression results in real-time PCR. Nucleic Acids Res..

[83] Vandesompele J (2002). Accurate normalization of real-time quantitative RT-PCR data by geometric averaging of multiple internal control genes. Genome Biol..

[84] Yuan R, Vos PM, Cooperberg PL (2006). Computer-aided detection in screening CT for pulmonary nodules. Am. J. Roentgenol..

[85] Challis GL, Ravel J, Townsend CA (2000). Predictive, structure-based model of amino acid recognition by nonribosomal peptide synthetase adenylation domains. Chem. Biol..

[86] Konz D, Marahiel MA (1999). How do peptide synthetases generate structural diversity?. Chem. Biol..

[87] Stachelhaus T, Marahiel MA (1995). Modular structure of genes encoding multifunctional peptide synthetases required for non-ribosomal peptide synthesis. FEMS Microbiol. Lett..

