# Supplementary material for: Identification of non-ribosomal peptide synthetase in Ganoderma boninense Pat. that was expressed during the interaction with oil palm
Source: Sci Rep. 2021 Aug 11;11:16330. doi: 10.1038/s41598-021-95549-8 (PMC8358039; doi:10.1038/s41598-021-95549-8)
Supplement: Supplementary file 1 — Supplementary Information 1. [file 41598_2021_95549_MOESM1_ESM.pdf]

Supporting Information

Identification of non-ribosomal peptide synthetase in *Ganoderma boninense* Pat. that was expressed during the interaction with oil palm (*Elaeis guineensis* Jacq.)

Neda Shokrollahi, Chai-Ling Ho, Nur Ain Izzati Mohd Zainudin[c], Mohd Aswad Bin Abul Wahab, Mui-Yun Wong

Table of Contents

Tables		Page
Table S 1	BLASTx result for the predicted genes by SeMPI v2 in *G. boninense* strain Nj3 compared with *G. sinense*	1
Table S 2	BLASTx result for the predicted genes by SeMPI v2 in *G. boninense* strain G3 compared with *G. sinense*	1
Table S 3	BLASTx result for NRPS region predicted by antiSMASH in *G. boninense* Strain NJ3 with E Value (0) and percent identity above 50%	4
Table S 4	BLASTx result for NRPS region predicted by antiSMASH in *G. boninense* Strain G3 with E Value (0) and percent identity above 50%	4
Table S 5	BLASTx result for NRPS region predicted by SeMPI v2 in *G. boninense* Strain Nj3 (Acc no: LFMK01009681.1) with E Value (0) and percent identity above 50%	5
Table S 6	BLASTx result for NRPS region predicted by SeMPI v2 in *G. boninense* Strain G3 (Acc No: PJEW02000037.1) with E Value (0) and percent identity above 50%	7
Table S 7	The difference of nuclides in the *GbNRPS* from strain UPMGb001compared to strains NJ3 and G3 obtained from alignment by using Clustal Omega	12
Table S 8	Percent identity matrix created by Clustal 2.1 to show the similarity of sequences among UPMGb001, NJ3 and G3 relative to each other	15
Table S 9	Sequence of oligonucleotide primers of NRPS region	15
Table S 10	The external signs and symptoms of BSR disease on oil palm with scale 0-4	16
Table S 11	Primer sequences used in qPCR	17

Blast hits for putative *Ganoderma boninense* strain Nj3 and G3 (Accession Number: LFMK00000000[1] and PJEW00000000[2], respectively) NRPS biosynthetic gene clusters predicted with antiSMASH. For confirmation of the antiSMASH results, we utilized SeMPI v2 (http://sempi.pharmazie.uni-freiburg.de)[3]. For this purpose, genes that were predicted as NRPS genes by SeMPI v2, were retrieved and submitted to the BLASTx server at National Centre for Biotechnology Information (NCBI, ftp://ftp.ncbi.nih.gov/blast/) for identification. The similarity of the genes was observed with *G. sinense* in the below table.

Table S 1 BLASTx result for the predicted genes by SeMPI v2 in *G. boninense* strain Nj3 compared with *G. sinense*

Gene name	Accession number	Location on the contig(bp)	E value	Max Identity	Accession
Transporter	LFMK01000773.1	7868- 8066	1e-21	93.94 %	PIL31438.1
hypothetical protein GSI_06140	LFMK01000773.1	8777- 9338	1e-87	96.82 %	PIL31438.1
hypothetical protein GSI_06140	LFMK01000773.1	9654- 10182	4e- 100	89.77 %	PIL31438.1
Transporter	LFMK01004971.1	20- 242	1e-12	55.41 %	PIL25981.1
Transporter	LFMK01004971.1	869- 1040	1e-22	90.38 %	PIL25981.1
Transporter	LFMK01004971.1	1100- 1205	2e-11	93.10 %	PIL25981.1
NRPS	LFMK01009681.1	28767- 28974	3e-29	80.88 %	PIL24012.1
NRPS	LFMK01009681.1	29403- 29739	2e-67	92.86 %	PIL24012.1
NRPS	LFMK01009681.1	29739- 30843	9e-32	96.49 %	PIL24012.1
hypothetical protein GSI_06336	LFMK01001091.1	4527- 5142	2e-123	94.00 %	PIL31634.1
hypothetical protein GSI_06336	LFMK01001091.1	5312- 5633	1e-65	97.20 %	PIL31634.1
hypothetical protein GSI_06329	LFMK01001032.1	34077- 34689	1e-125	97.00 %	PIL31627.1

Table S 2 BLASTx result for the predicted genes by SeMPI v2 in *G. boninense* strain G3 compared with *G. sinense*

**Gene name**	**Accession number**	**Location on the contig(bp)**	**E value**	**Max Identity**	**Accession**
Transporter	PJEW02000493.1	202154- 202352	1e-21	93.94 %	PIL31438.1
Transporter	PJEW02000493.1	203064- 203625	1e-87	96.82 %	PIL31438.1
Hypothetical protein GSI_10830	PJEW02000012.1	1225308-1225518	3e-36	98.57 %	PIL27678.1

Hypothetical protein GSI_10830	PJEW02000012.1	1225972-1226692	2e-155	95.83 %	PIL27678.1
Hypothetical protein GSI_10830	PJEW02000012.1	1226767-1227436	2e-145	97.31 %	PIL27678.1
Transporter	PJEW02000032.1	44496- 44637	1e- 20	95.74 %	PIL26715.1
NRPS	PJEW02000037.1	107383- 107590	5e- 38	100%	PIL24012.1
NRPS	PJEW02000037.1	108019- 108355	2e- 67	92.86 %	PIL24012.1
NRPS	PJEW02000037.1	109261- 109459	9e- 32	86%	PIL24012.1
Transporter	PJEW02000044.1	46363- 46789	2e- 84	91.55 %	PIL22879.1
Transporter	PJEW02000058.1	62411- 62966	9e- 120	94.59 %	PIL25731.1
Transporter	PJEW02000065.1	99329-99686	3e- 63	91.67 %	PIL22879.1
Transporter	PJEW02000077.1	49284- 49491	2e- 41	98.55 %	PIL24412.1
Transporter	PJEW02000092.1	170109- 170325	3e- 18	63.77 %	PIL29057.1
Transporter	PJEW02000092.1	171680- 171992	7e- 56	92.31 %	PIL29057.1
Hypothetical protein GSI_14567	PJEW02000110.1	195796- 196276	1e-91	93.79 %	PIL23257.1
Hypothetical protein GSI_14567	PJEW02000110.1	196531- 196924	1e-79	96.85 %	PIL23257.1
Transporter	PJEW02000136.1	263690- 264194	1e- 67	80.36 %	PIL25731.1
Hypothetical protein GSI_06140	PJEW02000308.1	39531- 39873	1e- 56	92.11 %	PIL31439.1
Transporter	PJEW02000177.1	344298- 344601	3e-54	89.11 %	PIL29048.1
Transporter	PJEW02000312.1	56365- 56539	3e-23	90.57 %	PIL25981.1
Transporter	PJEW02000312.1	56654- 56945	5e- 59	95.88 %	PIL25981.1
Transporter	PJEW02000312.1	57163- 57619	5e- 43	68.42 %	PIL25981.1
Transporter	PJEW02000190.1	136941- 137052	1e-13	85.71 %	PIL27096.1
Transporter	PJEW02000321.1	150971- 151070	7e- 14	100%	PIL22505.1
Transporter	PJEW02000321.1	152484- 152694	2e- 31	90.0%	PIL22505.1
Transporter	PJEW02000321.1	347046- 347253	1e-38	88. 24%	PIL22580.1
Transporter	PJEW02000333.1	176923- 177097	1e-30	93.10 %	PIL24412.1

Hypothetical protein GSI_06336	PJEW02000222.1	185608- 185929	1e- 65	97.20 %	PIL31634.1
Hypothetical protein GSI_06336	PJEW02000222.1	186099- 186714	3e-121	92.68 %	PIL31634.1
Transporter	PJEW02000340.1	37920- 38304	2e- 78	92.19 %	PIL27096.1
Transporter			1e-64	79.37 %	PIL29058.1
Transporter	PJEW02000340.1	39499- 39868	1e-70	87.80 %	PIL27096.1
Transporter	PJEW02000384.1	9503- 9824	3e- 56	97.20 %	PIL29057.1
Transporter	PJEW02000384.1	12375- 12738	1e-62	81.67 %	PIL27096.1
Transporter	PJEW02000384.1	13405- 13720	3e-71	97.14 %	PIL29058.1
Hypothetical protein GSI_10408	PJEW02000468.1	177566- 177890	5e-57	88.89 %	PIL27263.1
Transporter	PJEW02000472.1	36752- 36926	4e-26	98.11 %	PIL25981.1
Transporter	PJEW02000472.1	37041- 37335	1e-57	90.82 %	PIL25981.1
Transporter	PJEW02000472.1	37781- 38006	3e-18	81.33 %	PIL25981.1
Transporter	PJEW02000480.1	506372- 507053	7e-157	98.61 %	PIL28784.1
hypothetical protein GSI_14567	PJEW02000489.1	616147- 616729	3e-118	93.33 %	PIL23257.1
hypothetical protein GSI_14567	PJEW02000489.1	616878- 617232	7e-73	96.61 %	PIL23257.1

Table S 3 BLASTx result for NRPS region predicted by antiSMASH in *G. boninense* Strain NJ3 with E Value (0) and percent identity above 50%

Description	E Value	Max Identity	Accession No.
non-ribosomal peptide synthetase [*Ganoderma sinense* ZZ0214-1]	0	80%	PIL24012.1
peptide synthetase [*Dichomitus squalens* LYAD-421 SS1]	0	62%	XP_007361133.1
peptide synthetase [Polyporus brumalis]	0	64%	RDX56754.1
hypothetical protein PYCCODRAFT_1440485 [Trametes coccinea BRFM310]	0	62%	OSC97122.1
Nonribosomal peptide synthetase 2 [*Trametes pubescens*]	0	62%	OJT03537.1
hypothetical protein TRAVEDRAFT_27949 [*Trametes versicolor* FP-101664 SS1]	0	62%	XP_008036091.1
Nonribosomal peptide synthetase 2 [*Grifola frondosa*]	0	59%	OBZ75800.1

Table S 4 BLASTx result for NRPS region predicted by antiSMASH in *G. boninense* Strain G3 with E Value (0) and percent identity above 50%

Description	E Value	Max Identity	Accession No.
non-ribosomal peptide synthetase [*Ganoderma sinense* ZZ0214-1]	0	73%	PIL24012.1
peptide synthetase [*Dichomitus squalens* LYAD-421 SS1]	0	57%	XP_007361133.1
peptide synthetase [*Polyporus brumalis*]	0	64%	RDX56754.1
hypothetical protein PYCCODRAFT_1440485 [*Trametes coccinea* BRFM310]	0	62%	OSC97122.1
Nonribosomal peptide synthetase 2 [*Trametes pubescens*]	0	62%	OJT03537.1
hypothetical protein TRAVEDRAFT_27949 [*Trametes versicolor* FP-101664 SS1]	0	62%	XP_008036091.1
Nonribosomal peptide synthetase 2 [*Grifola frondosa*]	0	59%	OBZ75800.1

Table S 5 BLASTx result for NRPS region predicted by SeMPI v2 in *G. boninense* Strain Nj3 (Acc no: LFMK01009681.1) with E Value (0) and percent identity above 50**%**

**A: region: 28767- 28974 bp**			
Description	E Value	Max Identity	Accession No.
non-ribosomal peptide synthetase [*Ganoderma sinense* ZZ0214-1]	3e-29	80.88%	PIL24012.1
peptide synthetase [*Dichomitus squalens* LYAD-421 SS1]	4e-23	63.77%	XP_007361133.1
peptide synthetase [*Polyporus brumalis*]	2e- 20	59.42%	RDX56754.1
peptide synthetase [Lentinus tigrinus ALCF2SS1-6]	2e-20	60.87%	RPD66154.1
peptide synthetase [Polyporus arcularius HHB13444]	3e-20	57.97%	TFK91049.1
Nonribosomal peptide synthetase 2 [Trametes pubescens]	6e-17	55.07%	OJT03537.1
hypothetical protein TRAVEDRAFT_27949 [*Trametes versicolor* FP-101664 SS1]	3e-20	57.97%	TFK91049.1
**B: region: 29403- 29739 bp**			
Description	E Value	Max Identity	Accession No.
non-ribosomal peptide synthetase [*Ganoderma sinense* ZZ0214-1]	2e-67	92.86%	PIL24012.1
peptide synthetase [*Polyporus brumalis*]	1e-62	87.50%	RDX56754.1
peptide synthetase [*Polyporus arcularius* HHB13444]	1e-62	87.50%	TFK91049.1
peptide synthetase [*Lentinus tigrinus* ALCF2SS1-6]	4e-61	86.61%	RPD66154.1
hypothetical protein BN946_scf184785.g3 [Trametes cinnabarina]	1e-59	84.82%	CDO69498.1
peptide synthetase [*Dichomitus squalens*]	7e-59	84.82%	TBU50798.1
Nonribosomal peptide synthetase 2 [*Trametes pubescens*]	8e-58	82.14%	OJT03537.1
Nonribosomal peptide synthetase 2 [*Grifola frondosa*]	4e-57	82.14%	OBZ75800.1
siderophore biosynthesis enzyme [*Gelatoporia subvermispor*a B]	3e-54	77.68%	EMD38714.1
NRPS [*Termitomyces* sp. T32_za158]	1e-47	73.21%	KAG6898366.1

peptide synthetase [*Pholiota conissans*]	2e-47	72.32%	KAF9483747.1
Nonribosomal peptide synthetase 2 [*Hypsizygus marmoreus*]	3e-47	73.21%	RDB21442.1
Ferrichrome siderophore peptide synthetase [*Termitomyces* sp. T153]	9e-47	71.43%	KAG5736552.1
Ferrichrome siderophore peptide synthetase [Termitomyces sp. J132]	2e-46	71.43%	KNZ71811.1
peptide synthetase [*Lepista nuda*]	2e-46	67.86%	KAF9464762.1
nonribosomal peptide synthetase [*Hydnomerulius pinastri* MD-312]	2e-46	69.64%	KIJ69297.1
NRPS protein [*Sphagnurus paluster*]	5e-46	70.54%	KAG5652249.1
hypothetical protein CVT24_007726 [*Panaeolus cyanescens*]	6e-46	69.64%	PPR03610.1
hypothetical protein BDR05DRAFT_923562 [*Suillus weaverae*]	6e-46	69.64%	KAG2349803.1
nonribosomal peptide synthetase 12 [*Coprinopsis cinerea*]	8e-46	70.54%	KAG2020241.1
hypothetical protein BDR04DRAFT_1042778 [*Suillus decipiens*]	1e-45	70.54%	KAG2078239.1
hypothetical protein HYPSUDRAFT_63026 [*Hypholoma sublateritium* FD-334 SS-4]	1e-45	70.54%	KJA27343.1
peptide synthetase [*Coprinopsis cinerea* okayama7#130]	1e-45	70.54%	XP_001833231.2

C: region: 29739- 30843bp

Description	E Value	Max Identity	Accession No.
non-ribosomal peptide synthetase [*Ganoderma sinense* ZZ0214-1]	9e-32	96.49%	PIL24012.1
peptide synthetase [*Dichomitus squalens*]	1e-25	80.70%	TBU50798.1
peptide synthetase [*Dichomitus squalens* LYAD-421 SS1]	1e-25	80.70%	XP_007361133.1
peptide synthetase [*Polyporus brumalis*]	2e-21	71.93%	RDX56754.1
peptide synthetase [*Polyporus arcularius* HHB13444]	2e-21	71.93%	TFK91049.1
hypothetical protein PYCCODRAFT_1440485 [*Trametes coccinea* BRFM310]	5e-21	63.16%	OSC97122.1
hypothetical protein BN946_scf184785.g3 [Trametes cinnabarina]	2e-19	57.89%	CDO69498.1

hypothetical protein D9756_001510 [*Leucoagaricus leucothites*]	1e-16	56.14%	KAF5357820.1
peptide synthetase [*Lepista nuda*]	6e-16	54.39%	KAF9464762.1
hypothetical protein TRAVEDRAFT_27949 [Trametes versicolor FP-101664 SS1]	2e-15	57.89%	XP_008036091.1
hypothetical protein D9613_003107 [*Agrocybe pediades*]	6e-15	54.39%	KAF4614482.1
NRPS protein [Sphagnurus paluster]	7e-15	52.54%	KAG5652249.1
putative NRPS-like protein biosynthetic cluster [*Tephrocybe* sp. NHM501043]	9e-15	52.63%	KAG6845338.1
peptide synthetase [Coprinopsis cinerea okayama7#130]	1e-14	57.89%	XP_001833231.2
nonribosomal peptide synthetase 12 [*Coprinopsis cinerea*]	1e-14	57.89%	KAG2020241.1
hypothetical protein OBBRIDRAFT_790288 [*Obba rivulosa*]	2e-14	54.39%	OCH93414.1
peptide synthetase [*Cyathus striatus*]	2e-14	56.14%	KAF9013880.1
peptide synthetase [*Agrocybe pediades*]	3e-14	52.63%	KAF9564589.1
peptide synthetase [*Crassisporium funariophilum*]	3e-14	50.88%	KAF8163357.1
siderophore biosynthesis enzyme [*Gelatoporia subvermispora* B]	3e-14	52.63%	EMD38714.1
RecName: Full=Nonribosomal peptide synthase NPS2; Short=NPS2; Short=NRPS 2; Alt Name: Full=Type VI siderophore synthetase NPS2 [*Gelatoporia subvermispora* B]	3e-14	52.63%	A0A248AFK6.1
Nonribosomal peptide synthetase 2 [Hypsizygus marmoreus]	3e-14	50.88%	RDB21442.1
peptide synthetase [*Coprinopsis marcescibilis*]	4e-14	52.63%	TFK29988.1
Ferrichrome siderophore peptide synthetase [*Termitomyces* sp. J132]	4e-14	52.63%	KNZ71811.1
NRPS protein [*Termitomyces* sp. Mn162]	4e-14	52.63%	KAG5342803.1
Ferrichrome siderophore peptide synthetase [*Termitomyces* sp. T153]	4e-14	52.63%	KAG5736552.1
Ferrichrome siderophore peptide synthetase [*Termitomyces* sp. T112]	4e-14	52.63%	KAG5727948.1
hypothetical protein D9619_005643 [*Psilocybe* cf. subviscida]	5e-14	52.63%	KAF5330326.1
NRPS protein [*Asterophora parasitica*]	9e-14	52.63%	KAG5645251.1
peptide synthetase [*Coprinellus micaceus*]	2e-13	50.88%	TEB36377.1

Table S 6 BLASTx result for NRPS region predicted by SeMPI v2 in *G. boninense* Strain G3 (Acc No: PJEW02000037.1) with E Value (0) and percent identity above 50%

**A: region: 107383- 107590 bp**			
Description	E Value	Max Identity	Accession No.
non-ribosomal peptide synthetase [*Ganoderma sinense* ZZ0214-1]	3e-30	82.35%	PIL24012.1
peptide synthetase [*Dichomitus squalens*]	3e-24	65.22%	TBU50798.1

peptide synthetase [*Dichomitus squalens*]	4e-24	65.22%	TBU35961.1
peptide synthetase [*Dichomitus squalens*]	5e-24	65.22%	TBU65805.1
peptide synthetase [*Dichomitus squalens* LYAD-421 SS1]	5e-24	65.22%	XP_007361133.1
peptide synthetase [*Lentinus tigrinus* ALCF2SS1-6]	1e-20	60.87%	RPD66154.1
peptide synthetase [*Polyporus brumalis*]	1e-20	59.42%	RDX56754.1
peptide synthetase [*Polyporus arcularius* HHB13444]	3e-20	57.97%	TFK91049.1
Nonribosomal peptide synthetase 2 [*Trametes pubescens*]	7e-18	56.52%	OJT03537.1
hypothetical protein TRAVEDRAFT_27949 [*Trametes versicolor* FP-101664 SS1]	2e-17	55.07%	XP_008036091.1
hypothetical protein PYCCODRAFT_1440485 [*Trametes coccinea*BRFM310]	9e-16	48.53%	OSC97122.1
hypothetical protein BN946_scf184785.g4 [*Trametes cinnabarina*]	2e-15	51.56%	CDO69499.1

B: region: 108019- 108355 bp

Description	E Value	Max Identity	Accession No.
non-ribosomal peptide synthetase [*Ganoderma sinense* ZZ0214-1]	2e-67	92.86%	PIL24012.1
peptide synthetase [*Polyporus brumalis*]	1e-62	87.50%	RDX56754.1
peptide synthetase [*Polyporus arcularius* HHB13444]	1e-62	87.50%	TFK91049.1
peptide synthetase [*Lentinus tigrinus* ALCF2SS1-6]	4e-61	86.61%	RPD66154.1
hypothetical protein BN946_scf184785.g3 [*Trametes cinnabarina*]	1e-59	84.82%	CDO69498.1
hypothetical protein PYCCODRAFT_1440485 [*Trametes coccinea* BRFM310]	1e-59	84.82%	OSC97122.1
peptide synthetase [*Dichomitus squalens*]	6e-59	84.82%	TBU35961.1
peptide synthetase [*Dichomitus squalens*]	7e-59	84.82%	TBU50798.1
Nonribosomal peptide synthetase 2 [*Trametes pubescens*]	8e-58	82.14%	OJT03537.1
peptide synthetase [*Dichomitus squalens* LYAD-421 SS1]	1e-57	83.93%	XP_007361133.1
peptide synthetase [*Dichomitus squalens*]	1e-57	83.93%	TBU65805.1
hypothetical protein TRAVEDRAFT_27949 [*Trametes versicolor* FP-101664 SS1]	2e-57	82.14%	XP_008036091.1
Nonribosomal peptide synthetase 2 [*Grifola frondosa*]	4e-57	82.14%	OBZ75800.1
hypothetical protein OBBRIDRAFT_790288 [*Obba rivulosa*]	1e-54	77.68%	OCH93414.1
RecName: Full=Nonribosomal peptide synthase NPS2; Short=NPS2; Short=NRPS 2;AltName: Full=Type VI siderophore synthetase NPS2 [*Gelatoporia subvermispora* B]	2e-54	77.68%	A0A248AFK6.1
siderophore biosynthesis enzyme [*Gelatoporia subvermispora* B]	3e-54	77.68%	EMD38714.1

peptide synthetase [*Crassisporium funariophilum*]	3e-48	74.11%	KAF8163357.1
NRPS [*Termitomyces* sp. T32_za158]	1e-47	73.21%	KAG6898366.1
peptide synthetase [*Pholiota conissans*]	2e-47	72.32%	KAF9483747.1
Nonribosomal peptide synthetase 2 [*Hypsizygus marmoreus*]	3e-47	73.21%	RDB21442.1
Ferrichrome siderophore peptide synthetase [*Termitomyces* sp. T153]	9e-47	71.43%	KAG5736552.1
Ferrichrome siderophore peptide synthetase [*Termitomyces* sp. T112]	9e-47	71.43%	KAG5727948.1
hypothetical protein EV702DRAFT_961001 [*Suillus placidus*]	1e-46	69.64%	KAG1782590.1
Ferrichrome siderophore peptide synthetase [*Termitomyces* sp. J132]	2e-46	71.43%	KNZ71811.1
peptide synthetase [Lepista nuda]	2e-46	67.86%	KAF9464762.1
nonribosomal peptide synthetase [*Hydnomerulius pinastri* MD-312]	2e-46	69.64%	KIJ69297.1
hypothetical protein C0995_003749 [Termitomyces sp. Mi166#008]	3e-46	70.54%	KAG6874203.1
peptide synthetase [*Pluteus cervinus*]	3e-46	70.54%	TFK76388.1
hypothetical protein BJ165DRAFT_1443685 [*Panaeolus papilionaceus*]	5e-46	71.43%	KAF9052883.1
NRPS protein [*Sphagnurus paluster*]	5e-46	70.54%	KAG5652249.1
hypothetical protein CVT24_007726 [Panaeolus cyanescens]	6e-46	69.64%	PPR03610.1
nonribosomal peptide synthetase 12 [*Coprinopsis cinerea*]	8e-46	70.54%	KAG2020241.1
hypothetical protein BDR04DRAFT_1042778 [*Suillus decipiens*]	1e-45	70.54%	KAG2078239.1
hypothetical protein HYPSUDRAFT_63026 [*Hypholoma sublateritium* FD-334 SS-4]	1e-45	70.54%	KJA27343.1
peptide synthetase [*Coprinopsis cinerea* okayama7#130]	1e-45	70.54%	XP_001833231.2
hypothetical protein JR316_003860 [*Psilocybe cubensis*]	1e-45	70.54%	KAG5171772.1
peptide synthetase [*Coprinellus micaceus*]	2e-45	68.75%	TEB36377.1
uncharacterized protein EDB91DRAFT_31035 [*Suillus paluster*]	2e-45	67.86%	XP_041184524.1
peptide synthetase [*Coprinopsis marcescibilis*]	2e-45	71.43%	TFK29988.1
uncharacterized protein HD556DRAFT_608125 [Suillus plorans]	2e-45	70.54%	XP_041166127.1
hypothetical protein D9756_001510 [Leucoagaricus leucothites]	3e-45	69.64%	KAF5357820.1
unnamed protein product [Agrocybe aegerita]	3e-45	69.64%	CAA7271648.1
peptide synthetase [Crucibulum laeve]	5e-45	70.54%	TFK42523.1

hypothetical protein EV424DRAFT_1369342 [*Suillus variegatus*]	6e-45	69.64%	KAG1832025.1
hypothetical protein M413DRAFT_438219 [*Hebeloma cylindrosporum* h7]	6e-45	71.43%	KIM49051.1
**C: region: 109261- 109459 bp**			
Description	E Value	Max Identity	Accession No.
non-ribosomal peptide synthetase [*Ganoderma sinense* ZZ0214-1]	9e-32	96.49%	PIL24012.1
peptide synthetase [*Dichomitus squalens*]	1e-25	80.70%	TBU35961.1
peptide synthetase [*Dichomitus squalens*]	1e-25	80.70%	TBU50798.1
peptide synthetase [*Dichomitus squalens* LYAD-421 SS1]	1e-25	80.70%	XP_007361133.1
peptide synthetase [*Dichomitus squalens*]	3e-25	80.70%	TBU65805.1
peptide synthetase [*Lentinus tigrinus* ALCF2SS1-6]	9e-24	75.44%	RPD66154.1
peptide synthetase [*Polyporus brumalis*]	2e-21	71.93%	RDX56754.1
peptide synthetase [*Polyporus arcularius* HHB13444]	2e-21	71.93%	TFK91049.1
hypothetical protein PYCCODRAFT_1440485 [*Trametes coccinea* BRFM310]	5e-21	63.16%	OSC97122.1
hypothetical protein BN946_scf184785.g3 [*Trametes cinnabarina*]	2e-19	57.89%	CDO69498.1
hypothetical protein D9756_001510 [Leucoagaricus leucothites]	1e-16	56.14%	KAF5357820.1
peptide synthetase [*Lepista nuda*]	6e-16	54.39%	KAF9464762.1
hypothetical protein TRAVEDRAFT_27949 [*Trametes versicolor* FP-101664 SS1]	2e-15	57.89%	XP_008036091.1
hypothetical protein D9613_003107 [*Agrocybe pediades*]	6e-15	54.39%	KAF4614482.1
NRPS protein [*Sphagnurus paluster*]	7e-15	52.54%	KAG5652249.1
putative NRPS-like protein biosynthetic cluster [Tephrocybe sp. NHM501043]	9e-15	52.63%	KAG6845338.1
peptide synthetase [*Coprinopsis cinerea* okayama7#130]	1e-14	57.89%	XP_001833231.2
nonribosomal peptide synthetase 12 [*Coprinopsis cinerea*]	1e-14	57.89%	KAG2020241.1
hypothetical protein OBBRIDRAFT_790288 [*Obba rivulosa*]	2e-14	54.39%	OCH93414.1
peptide synthetase [*Cyathus striatus*]	2e-14	56.14%	KAF9013880.1
peptide synthetase [*Agrocybe pediades*]	3e-14	52.63%	KAF9564589.1
peptide synthetase [*Crassisporium funariophilum*]	3e-14	50.88%	KAF8163357.1
siderophore biosynthesis enzyme [*Gelatoporia subvermispora* B]	3e-14	52.63%	EMD38714.1
RecName: Full=Nonribosomal peptide synthase NPS2; Short=NPS2; Short=NRPS 2;AltName:	3e-14	52.63%	A0A248AFK6.1

Full=Type VI siderophore synthetase NPS2 [*Gelatoporia subvermispora* B]			
Nonribosomal peptide synthetase 2 [*Hypsizygus marmoreus*]	3e-14	50.88%	RDB21442.1
peptide synthetase [*Coprinopsis marcescibilis*]	4e-14	52.63%	TFK29988.1
Ferrichrome siderophore peptide synthetase [*Termitomyces* sp. J132]	4e-14	52.63%	KNZ71811.1
NRPS protein [*Termitomyces* sp. Mn162]	4e-14	52.63%	KAG5342803.1
Ferrichrome siderophore peptide synthetase [*Termitomyces* sp. T153]	4e-14	52.63%	KAG5736552.1
Ferrichrome siderophore peptide synthetase [*Termitomyces* sp. T112]	4e-14	52.63%	KAG5727948.1
hypothetical protein D9619_005643 [*Psilocybe* cf. *subviscida*]	5e-14	52.63%	KAF5330326.1
hypothetical protein M413DRAFT_438219 [*Hebeloma cylindrosporum* h7]	6e-14	50.88%	KIM49051.1
NRPS protein [*Asterophora parasitica*]	9e-14	52.63%	KAG5645251.1

The DNA fragments were amplified from UPMGb001 strain and constructed into full-length *GbNRPS* based on the sequencing result from the first base company. The full-length *GbNRPS* of UPMGb001 strain was aligned with that of NJ3 and G3 and found to show some variations at position 1342,1345,1346, 1348, 1351-1355 ,1357-1364,2444, 2490-2493,2497,2503-2505,2507,2509-2510,2512-2514,2598-2600,2602,3020, 4041,5078,5081 and 7773. The different nucleotides in the related position were shown in Table S 7. Percent identity among strains, was created by Clustal 12.1, interpreted the sequence similarities of UPMGb001, NJ3 and G3 as demonstrated in Table S 8.

Table S 7 The difference of nuclides in the *GbNRPS* from strain UPMGb001compared to strains NJ3 and G3 obtained from alignment by using Clustal Omega

Position	Strain	nucleotide
1342	UPMGB001	A
	NJ3	C
	G3	C
1345-1346	UPMGB001	AC
	NJ3	CT
	G3	CT
1348	UPMGB001	C
	NJ3	T
	G3	T
1351-1355	UPMGB001	GCCAT
	NJ3	CGTCC
	G3	CGTCC
1357-1364	UPMGB001	ACGGAGTC
	NJ3	TAACGAGT
	G3	TAACGAGT
2045	UPMGB001	-*
	NJ3	-
	G3	A

2444	UPMGB001	T
	NJ3	C
	G3	C
2490-2493	UPMGB001	CTTT
	NJ3	G--C
	G3	GCGC
2497	UPMGB001	A
	NJ3	T
	G3	T
2502-2504	UPMGB001	ACG
	NJ3	GTA
	G3	GTA
2507	UPMGB001	C
	NJ3	G
	G3	G
2509-2510	UPMGB001	AC
	NJ3	CT
	G3	CT
2512-2514	UPMGB001	CTC
	NJ3	ACA
	G3	ACA
2598-2600	UPMGB001	TCG
	NJ3	CAC
	G3	CAC
2602	UPMGB001	C

		NJ3	A
		G3	A
		UPMGB001	C
	3020	NJ3	G
		G3	G
		UPMGB001	K
	4041	NJ3	T
		G3	T
		UPMGB001	Y
	5078	NJ3	C
		G3	C
		UPMGB001	S
	5081	NJ3	G
		G3	G
		UPMGB001	A
7773		NJ3	-
		G3	A

*: indicates gaps introduced the sequence alignment

Table S 8 Percent identity matrix created by Clustal 2.1 to show the similarity of sequences among UPMGb001, NJ3 and G3 relative to each other

	UPMGb001	NJ3	G3
UPMGb001	100%	99.50%	99.48%
Nj3	99.50%	100%	99.95%
G3	99.48%	99.95%	100%

The primer design was done based on the overlapping; thus, the forward primer of the latter set was placed before the reverse primer of the previous set. All primers were analyzed using NCBI BLAST tool against the available database to ensure primer specificity. Table S7 listed the primer sequences.

Table S 9 Sequence of oligonucleotide primers of NRPS region

No.	Name of primer	Primer sequence 5'-3'	length	Amplicon Size
1	NRPS-GB1-F1	TTCAAGCGATGCTTCATAGG	20	444
	NRPS-GB1-R1	GACGGTAGGGGCCATGAT	18	
2	NRPS-GB2-F2	TCGAAAATTGTACGCACCTG	20	460
	NRPS-GB2-R2	CCGACAACATCGTCTTTTCC	20	
3	NRPS-GB3-F3	GCGTTCGATGTCCTTCAGAT	20	575
	NRPS-GB3-R3	AAGAGGTGTTGCCTGCACTT	20	
4	NRPS-GB4-F4	TAGGGTCTTCATTCGGTGCT	20	768
	NRPS-GB4-R4	GCCACGATCCTTTGTACCAT	20	
5	NRPS-GB5-F5	ACCATCGAGCTCTCGGAATA	20	334
	NRPS-GB5-R5	CTCCGATGAGTGGGACAAC	19	
6	NRPS-GB6-F6	GGAGAGGAGTTCTCGGAAGC	20	568
	NRPS-GB6-R6	CGTCTTCCAAGAGGGCATT	19	
7	NRPS-GB7-F7	TGTTTCAGGAGTGTCTTGTTCG	22	700
	NRPS-GB7-R7	TGGTCTCAGCGGCAGTATG	19	
8	NRPS-GB8-F8	ACTCGGTCAAGTTTGCGTGA	20	460
	NRPS-GB8-R8	TTATCCACGCCTATCCGATT	20	
9	NRPS-GB9-F9	AGACGGGTCTTGTTCGGTTT	20	531
	NRPS-GB9-R9	CTCCGCCTGGAAATGTTACT	20	
10	NRPS-GB10-F10	ATTTCCAGGCGGAGGTGTAG	20	700

	NRPS-GB10-R10	GTACGCTCGTCAGCCTCAGT	20	
11	NRPS-GB11-F11	GTTCCAGGAGCGTAACGTGT	20	489
	NRPS-GB11-R11	GATTTATGCAACGCCTGGAG	20	
12	NPRS-GB12-F12	CGGGGAAGGAAGGAAACTAA	20	575
	NPRS-GB12-R12	CGGTCTGGACCTCAATGTCT	20	
13	NRPS-GB13-F13	ATTGGTCCCCCGTCATAAG	19	499
	NRPS-GB13-R13	CAGACGATGGCACGGAAC	18	
14	NRPS-GB14-F14	GATGTTTGGCGAGAATGGTT	20	732
	NRPS-GB14-R14	TCACCCATTTACGGGTGTCT	20	
15	NRPS-GB15-F15	GATGAGGCTAGGGACAATGC	20	695
	NRPS-GB15-R15	GTTGCTACGTTCCGGTAAGC	20	
16	NRPS-GB16-F16	ATCGGTGACGCTTCCTCTAA	20	597
	NRPS-GB16-R16	CGCTCCCACAACCTACCAT	19	
17	NRPS-GB17-F17	AGGCGGTTATTGACATTTGG	20	598
	NRPS-GB17-R17	CGTTTGCTCAACCGTTCC	18	

Table S 10 The external signs and symptoms of BSR disease on oil palm with scale 0-4

Disease Class	Signs and symptoms of infection
0	Healthy plants with green leaves without the appearance of fungal mycelium on any part of plants
1	The appearance of white fungal mass on any part of plants, with or without chlorotic leaves
2	The appearance of basidioma on any part of plants with chlorotic leaves (1-3 leaves)
3	Formation of basidioma on any part of plants with chlorotic leaves (>3 leaves)
4	Formation of well-developed basidioma and the plants dried

The primers used in qPCR for gene expression were listed in table 5

Table S 11 Primer sequences used in qPCR

Gene	primer	Type of Primer	Primer Sequence (5'⟶3')	Product size[bp]	Reference
eEF2	GER 1	HKG[1]	F-TGGTCAAGAACATCCGTAT	173	[4]
	GER 2		R-CGCTAACAAAGACAAGGG		
α tubulin	GTR 7	HKG	F-GCACCGACTCTGGTGATGCT	100	[4]
	GTR 8		R-GATAGGCTATGGTCGCGAAG		
NRPS-C	C-forward	GSP[2]	F-CGAAGGTCCCACCCGTCCGA	136	
	C-reverse		R-TACGCGCGCTCGAGGTCTTG		

[1]HKG: Housekeeping gene (Reference gene), [2] GSP: Gene specific Primer

Reference

1 Mercière, M. *et al.* Identification and development of new polymorphic microsatellite markers using genome assembly for Ganoderma boninense, causal agent of oil palm basal stem rot disease. *Mycological Progress* **14**, 103 (2015).

2 Utomo, C. *et al.* Draft genome sequence of the phytopathogenic fungus *Ganoderma boninense*, the causal agent of basal stem rot disease on oil palm. *Genome Announc.* **6**, e00122-00118 (2018).

3 Zierep, P. F., Ceci, A. T., Dobrusin, I., Rockwell-Kollmann, S. C. & Günther, S. SeMPI 2.0—A Web Server for PKS and NRPS Predictions Combined with Metabolite Screening in Natural Product Databases. *Metabolites* **11**, 13 (2021).

4 Lim, F.-H., Rasid, O. A., Idris, A. S. & Parveez, G. K. A. MOLECULAR CLONING OF Ganoderma boninense HOG1-TYPE MITOGEN-ACTIVATED PROTEIN KINASE (MAPK) cDNA AND TRANSCRIPTIONAL RESPONSE TO SALINITY STRESS. *Journal of Oil Palm Research* **30**, 380-389 (2018).
